# Acceleration of bone repairation by BMSCs overexpressing NGF combined with NSA and allograft bone scaffolds

**DOI:** 10.1186/s13287-024-03807-z

**Published:** 2024-07-02

**Authors:** Ying Ji, Yongkang Mao, Honghu Lin, Ye Wang, Peishuai Zhao, Yong Guo, Lantao Gu, Can Fu, Ximiao Chen, Zheng Lv, Ning Wang, Qiang Li, Chaoyong Bei

**Affiliations:** 1 Department of Orthopaedics, Affiliated Hospital of Guilin Medical University, 15 Lequn Road, Guilin, 541001 China; 2 Department of Biomedical Engineering, School of Intelligent Medicine and Biotechnology, Guilin Medical University, 1 Zhiyuan Road, Guilin, 541199 China; 3 Key Laboratory of Medical Biotechnology and Translational Medicine, Guilin Medical University, 1 Zhiyuan Road, Guilin, 541199 China; 4 Department of Radiology, Affiliated Hospital of Guilin Medical University, 15 Lequn Road, Guilin, 541001 China

**Keywords:** BMSCs, NGF, P75NTR, Pyroptosis, Bone tissue engineering, Bone regeneration

## Abstract

**Background:**

Repairation of bone defects remains a major clinical problem. Constructing bone tissue engineering containing growth factors, stem cells, and material scaffolds to repair bone defects has recently become a hot research topic. Nerve growth factor (NGF) can promote osteogenesis of bone marrow mesenchymal stem cells (BMSCs), but the low survival rate of the BMSCs during transplantation remains an unresolved issue. In this study, we investigated the therapeutic effect of BMSCs overexpression of NGF on bone defect by inhibiting pyroptosis.

**Methods:**

The relationship between the low survival rate and pyroptosis of BMSCs overexpressing NGF in localized inflammation of fractures was explored by detecting pyroptosis protein levels. Then, the NGF^+^/BMSCs-NSA-Sca bone tissue engineering was constructed by seeding BMSCs overexpressing NGF on the allograft bone scaffold and adding the pyroptosis inhibitor necrosulfonamide(NSA). The femoral condylar defect model in the Sprague–Dawley (SD) rat was studied by micro-CT, histological, WB and PCR analyses in vitro and in vivo to evaluate the regenerative effect of bone repair.

**Results:**

The pyroptosis that occurs in BMSCs overexpressing NGF is associated with the nerve growth factor receptor (P75NTR) during osteogenic differentiation. Furthermore, NSA can block pyroptosis in BMSCs overexpression NGF. Notably, the analyses using the critical-size femoral condylar defect model indicated that the NGF^+^/BMSCs-NSA-Sca group inhibited pyroptosis significantly and had higher osteogenesis in defects.

**Conclusion:**

NGF^+^/BMSCs-NSA had strong osteogenic properties in repairing bone defects. Moreover, NGF^+^/BMSCs-NSA-Sca mixture developed in this study opens new horizons for developing novel tissue engineering constructs.

**Supplementary Information:**

The online version contains supplementary material available at 10.1186/s13287-024-03807-z.

## Introduction

Clinical bone defects caused by trauma, bone tumors, and osteomyelitis have become a challenging problem in orthopedics. Currently, clinical treatment of the above bone defects is usually regenerative bone tissue repair by transplanting autologous, allogeneic, xenograft, and inorganic bone at the site of the bone defect, and different bone tissue materials have been widely used for treating complex bone defects [1–4]. However, the potential risk of infection, poor osteoinductivity, and host inflammatory response induced by biomaterials may adversely affect the bone regenerative repair outcome [5, 6]. Consequently, these issues limit their practical application. Numerous studies reveal that combining cells, growth factors, and biomaterials to construct bone tissue engineering facilitates bone defect repair and regeneration [7–9]. Therefore, developing a new bone tissue engineering material with anti-inflammatory and degradability functions, such as biocompatibility and strength, to promote bone defect repair has become a hot spot of research.

Stem cells have strong self-renewal and multidirectional differentiation capabilities and have great potential for application in bone tissue engineering [10, 11]. Bone marrow mesenchymal stem cells (BMSCs) are pluripotent stem cells in the bone marrow [12]. BMSCs maintain their multidirectional differentiation potential in vitro and differentiate into bone tissues when transplanted in vivo [13]. BMSCs are widely used in clinical research and therapy due to these biological properties and have become an important transplantation cell for tissue engineering [14]. Nerve growth factor (NGF) is a nerve cell growth regulator [15], nourishing and promoting nerve protrusion growth, and it can regulate and promote BMSCS osteogenesis [16]. Studies have been shown, NGF promotes the long bone development innervated by sensory nerves and plays an important role in fracture healing [17, 18]. Therefore, NGF as a bone graft to construct bone tissue engineering materials holds great promise.

Although NGF has the potential to provide cutting-edge solutions for bone tissue engineering, challenges remain in stabilizing its ability to promote osteogenesis of BMSCs. Our preliminary research shows that, although NGF has enhanced biological activity in its ability to regulate the BMSC osteogenesis, inflammatory factors around the grafted cells have high expression during the process of NGF as a bone graft to promote fracture healing, and the nerve growth factor receptor (P75NTR) content was persistently increased in tissues of fracture non-healing [19]. P75NTR is a low affinity transmembrane receptor for NGF. Studies have shown its association with cell and tumour migration and invasion [20, 21]. We found that silencing the P75NTR gene enhances osteogenic differentiation of rat BMSCs [22]. A recent study has revealed that P75NTR can activate the NF-κB signaling pathway to aggravate cell damage [23, 24]. The NF-κB signaling pathway is closely linked to the NLRP3-mediated inflammatory response, and increased NLRP3 induces pyroptosis. Therefore, We suspect that the reduced survival of BMSCs overexpressing NGF may be related to P75NTR-induced inflammation, which leads to pyroptosis via the NF-κB signaling pathway. Pyroptosis is a highly regulated programmed cell death process [25], manifested by activating NF-κB by pathogens or stressors [26]. This leads to activation of the NLRP3 inflammasome, and caspase-1, then releasing pro-inflammatory mediator IL-1β and aggregating GSDMD. Finally, the rapid dissolution of plasma membrane causes cell death [27]. Additionally, studies have shown that pharmacological or genetic interventions can inhibit cellular pyroptosis [28, 29]. Therefore, improving the survival of BMSC overexpressing NGF and elucidating the related mechanism become the key research questions.

Recently, necrosulfonamide was identified as a potent inhibitor of the pore-forming protein gasdermin D (GSDMD), and it can inhibit the release of pro-inflammatory mediators, such as IL-1β [30]. In this study, it was verified that the low survival rate of BMSCs overexpressing NGF in the local inflammatory response of fractures was closely related to pyroptosis. In vitro, we compared the effects of NGF^+^/BMSCs, BMSCs-NSA, and NGF^+^/BMSCs-NSA on pyroptosis of BMSCs. And the final analysis showed that the pyroptosis reaction of BMSCs overexpressing NGF could be blocked by NSA. In vivo, we implanted BMSCs containing NGF^+^/BMSCs, BMSCs-NSA, and NGF^+^/BMSCs-NSA on the allogeneic bone to construct bone tissue-engineered grafts. And we transplanted them into the rat femoral condylar defects, and monitored new bone formation in a distal femoral defect model. The underlying mechanism of osteogenic differentiation of BMSCs overexpressing NGF was further investigated. We demonstrated that osteoblastic differentiation of BMSCs overexpressing NGF in the inflammatory response of fractures is closely related to pyroptosis. At the same time, overexpression of NGF combined with NSA can inhibit this pyroptosis and improve the survival rate of BMSCs, and enhance the osteogenic ability of BMSCs overexpression of NGF. This study can provide guidance for the design of new bone tissue engineering materials.

## Materials and methods

### In Vitro Study

#### Origin, culture and identification of BMSCs

BMSCs were purchased from Cyagen (RASMX-01001; Guangzhou, China). Low glucose dulbecco’s modified Eagle medium (L-DMEM) (Gibco, Billings, USA) supplemented with 10% fetal bovine serum (FBS; Gibco) and 1% penicillin–streptomycin solution (Gibco) was used as the medium. The medium was regularly changed after every 3 days. And the cells were cultured at 37 °C in a humidified atmosphere consisting of 5% CO2 in incubator.When the cultures approached 70–80% confluence, the cells were serially subcultured through passaging.

The purchased BMSCs identification quality test is qualified, and an additional file shows this in more detail [see Additional file 1]. In addition, the cell immunophenotyping of BMSCs was evaluated using flow cytometry. Briefly, the cells were suspended in phosphate-buffered saline (PBS; Solarbio, China) at a concentration of 10^6^ cells/mL. Thereafter, the cells were incubated with antibodys, including CD 34-FITC (PA5-85,917; eBioscience, San Diego, CA, USA), CD44-PE (103,007; Biolegend, San Diego, CA, USA), CD90-PE (MA1-80,650; eBioscience), anti-rat CD29-PE (102,207; Biolegend). The expression level of the antigen markers was analyzed through a BD FACSCanto (BD Biosciences, San Jose, CA). Collected data were analyzed using flow cytometry software (FlowJo V10, Flowjo, LLC, Ashland, OR, USA). 

#### Lentiviral transduction and characterization

The cells were transfected using an NGF overexpressed lentiviral vector, a P75NTR overexpressed lentiviral vector and a P75NTR knockdown lentiviral vector (GENECHEM, Shanghai, China) BMSCs were seeded into six-well plate at a density of 8.0 × 104 cells/well. When the cells were fused to 70–80%, the lentiviral vector infection solution was configured according to the manufacturer's instructions. There-after, the multiplicity of infection was set to 50, and BMSCs were transfected with NGF overexpressing lentiviral vector in the presence of HiTransG P infection enhancement solution. After culturing for 16 h, the infection solution was removed. Then, the fluorescent protein expression of the genes was observed using an inverted fluorescence microscope after three days.

#### Determination of NSA optimal concentration

The BMSCs were treated by necrosulfonamide (HY-100573; MCE, NJ, USA) with a concentration gradient of 0, 0.5, 1.0, 1.5, 2.0, and 3.0 μM, and normal L-DMEM medium was used as a control. Briefly, BMSCs were seeded in 96-well plates at a density of 5000 cells per well. After removing the medium from each well, MTT (5 mg/ml, Sigma-Aldrich, St. Louis, MO, USA) was added to each well and cultured for 3 h. There-after, the MTT was removed and 200 μL of DMSO (Sigma-Aldrich) were added to each well to dissolve the crystals. Then, the absorbance was measured at 490 nm using an enzyme marker (Synergy HT). Based on the above results, the optimal concentration of NSA that best promotes BMSC proliferation was prepared, and the following experiments were performed at this concentration.

#### Alizarin red S (ARS) staining

BMSCs were seeded into 0.1% gelatin-coated six-well plates. After culturing the cells to 70% fusion, the standard medium was replaced with Rat BMSC Osteogenic Induction and Differentiation Medium (RAXMX-90021; Cyagen, Guangzhou, China) and changed every three days, as described previously [31]. The kit included OriCell® Basal Medium For Cell Culture (177 mL; BLDM-03011), OriCell® Fetal Bovine Serum (Superior-Quality) (20 mL; FBSSR-01021), OriCell® Supplement For Rat BMSC Osteogenic Differentiation (3 mL; RAXMX-04021), Alizarin Red S (10 mL; ALIR-10001 and Gelatin (10 mL; GLT-11301).After two and four weeks of osteogenic induction, the samples were fixed with a 4% paraformaldehyde for 30 min and stained with alizarin red for 5 min, respectively. Finally, calcium deposition images were taken using a microscope (Olympus Corporation). The cells were collected after osteogenic induction two weeks for Western blot experiments.

#### Western blot assay

BMSCs were seeded into six-well plates at a density of 80% and grouped according to the experimental design. The medium was discarded after treatment, and the wells were washed three times with cold PBS. The RIPA buffer (P0013B; Beyotime Biotechnology, China) containing a mixture of protease and phosphatase inhibitors was used to isolate the proteins. After that, centrifugation at 12,000 × g for 15 min at 4 °C, and the supernatant was collected. The Protein was quantified using a BCA protein assay kit (P0010S; Beyotime).

Proteins (20 μg/lane) were separated by SDS-PAGE (10% gel) and then transferred to polyvinylidene fluoride (PVDF) membranes (IPVH00010; Millipore, USA). Then the membranes were blocked with a sealing solution (P0023B-100 ml; Beyotime) at 25 °C for 1 h. The proteins were washed thrice with TBST for 10 min each and incubated with primary antibodies overnight at 4 °C. Table 1 presents the list of the primary antibodies. The membranes were washed with TBST and incubated with the corresponding secondary antibody (1;5,000, Proteintech, Wuhan, China) for 1 h. After rinsing with TBST for three times, the signal was detected with an chemiluminescence reagent (WBKLS0100; Millipore) and used the ECL program (Bio-Rad, USA). Finally, the results were scanned and analyzed using a gel imaging system (ChemiDoc™ XRS + System, Bio-rad). Image J was used to analyze the gray level. The experiment was repeated three times.

**Table 1:** The primary antibody information of this study

Primary antibody Name	Company and catalog	Dilution ratio	Molecular weight (kDa)
P75NTR	Abcam, ab52987	1:2,000	75
GSDMD	Abcam, ab219800	1:2,000	53
NLRP3	Abcam, ab263899	1:1,000	118
IL-1β	Abcam, ab234437	1:1,000	31
OCN	thermo Fisher Scientific, 33–5400	1:1,000	14
β-Actin	Proteintech, 81,115–1-RR	1;5,000	42
GAPDH	Proteintech, 10,494–1-AP	1;5,000	36

#### Physicochemical properties of the scaffolds

Allogeneic bone scaffolds (Cojoing, beijing, China) with the three-dimensional mesh structure of natural bone tissue were prepared. The scaffold chemical structure was determined by Fourier transform infrared spectroscopy (FTIR; Nicolet IS10) and hydrogen proton nuclear magnetic resonance spectroscopy (1H NMR; Bruker AVANCE III 600 M). The scaffold morphological structure was observed using scanning electron microscopy (SEM; Zeiss Gemini SEM 300, Germany).

#### Cell attachment study

BMSCs were seeded on allogeneic bone scaffolds and co-cultured for three days. The surface morphology of cells grown on the scaffolds was assessed using SEM. After 72 h of cell inoculation, the cell scaffold constructs were washed thrice with PBS and fixed in a multi-step method. Initially, the constructs were fixed with 2.5 wt% (Solarbio) for 2 h. Then, the samples were dehydrated in ethanol with concentration gradients of 50%, 70%, 80%, 90%, 100% I, and 100% II. Finally, the scaffolds were vacuum dried, coated with gold sputtering (Quorum Technologies Ltd Desk sputtercoater, UK), and observed using a Zeiss Gemini SEM 300 at an accelerating voltage of 15 kV.

### In Vivo study

#### Animal model design and surgical implantation

In this study, a rat model of critical-size femoral defects was made using 56 male SD rats aged 12 weeks. The animals were purchased from SJA Laboratory Animal Co., Ltd (Hunan, China) and individually housed in captivity in the Experimental Animal Center of Guilin Medical College with controlled temperature and light cycles (24 °C and 12/12 light cycle). And the animals with free access to standard diet and drinking water. All animal experiments were conducted according to protocols approved by the Animal Ethics and Use Committee of Guilin Medical College (GLMC-IACUC-20241012), and the study protocols adhere to the ARRIVE guidelines.

The fifty-six rats were randomly divided into four groups as follows (n = 14 per group): the BMSCs-Sca group, the BMSCs-NSA-Sca group, the NGF^+^/BMSCs-NSA-Sca group, the NGF^+^/BMSCs-Sca group. The rats were induced with 3% (w/v) pentobarbital salt solution (0.1 mL/100 g; Ceva, France) and de-haired, disinfected, and scarfed around the right femoral condyle. The subcutaneous tissues and muscles were incised layer-by-layer to expose the lower end of the right femur. A defect with a diameter of 4.5 mm and a depth of 5 mm was created by a dental microdrill (Thomas, France) to ensure a defined critical dimension. The incision did not penetrate the joint cavity to protect the articular cartilage. Then, allograft bone implanted with NGF^+^/BMSCs, NGF^+^/BMSCs-NSA, BMSCs-NSA, and BMSCs alone was filled into the defect, the incision was routinely sutured, and the rats were allowed to move freely after the operation. The defects filled with allograft bone containing BMSCs alone were considered control groups. The femoral condyles were collected from 28 rats at four and eight weeks aftre surgery. Notably, the animals were given 3% (w/v) pentobarbital salt solution in order to minimalize the stress and suffering of animals. 15 min after administration of anaesthetics, animals were euthanized by the disruption of the spinal cord. Qne part was used for Quantitative real-time polymerase chain reaction (qRT-PCR) and Western blot; the other part was fixed with 4% (w/v) paraformaldehyde at 4 °C for 24 h. The samples were processed, analyzed by Micro-Computed Tomography (Micro-CT), and decalcified with EDTA decalcification solution (pH 7.2) for three months for histological studies.

#### Micro-CT analysis

Micro-CT analysis was used to quantify the bone formation volume within the defect. Two and four weeks after surgery, the right femoral condyle was harvested and fixed with 4% paraformaldehyde. After processing, the samples were measured using a high-resolution 3D X-ray microscope (Zeiss Xradia 510 Versa) at 50 kV, 120 mA, and a pixel resolution of 9 µm. After scanning, a 3D image model was reconstructed using CTAN image analysis software. Based on a previous study [32], a hollow cylinder (ROI) with a diameter of 4.5 mm and a depth of 5 mm was selected to determine the percentage of bone volume/tissue volume (BV/TV %) to assess bone regeneration of the bone defect. Furthermore, the trabecular bone parameters analyzed and quantified to determine the trabecular number (Tb.N), trabecular separation (Tb.Sp), and trabecular thickness (Tb.Th).

#### RNA extraction and qRT-PCR

The pyroptosis and osteogenic gene expressions in animal tissues were analyzed by qRT-PCR. Fresh tissues were ground, and total RNA was extracted using the GeneJET™ RNA Purification Kit (K0732; Thermo Fisher Scientific, Warsaw, Poland, USA). Then, cDNA was synthesized using the RevertAid First Strand cDNA Synthesis Kit-LBID (K16225; Thermo) according to the manufacturer's instructions. After that, using a real-time fluorescent PCR instrument (Thermo, 7900HT Fast Real-Time PCR System), PCR was performed by PowerUp™ SYBR™ Green Master Mix (A25742; Thermo). Finally, the relative expression of the associated factors was calculated by the Comparison 2-ΔΔCt method. The primer sequences used in this study are listed in Table 2.

**Table 2:** Primers for qRT-PCR of this study

Gene name	Forward primer(5’-3’)	Reverse primer(5’-3’)
GSDMD	CCAGCATGGAAGCCTTAGAG	CAGAGTCGAGCACCAGACAC
IL-1β	TGACTTCACCATGGAACCCG	TCCTGGGGAAGGCATTAGGA
GAPDH	CTCATGACCACAGTCCATGC	TTCAGCTCTGGGATGACCTT

#### Western blot assay

Pyroptosis and osteogenic gene expression in animal tissues were analyzed using Western blot. After grinding the fresh tissue, and tissue proteins were extracted as described in previous protocol. Finally, the samples were scanned and analyzed using a gel imaging system (Bio-rad, ChemiDoc™ XRS + System, USA) and quantified using ImageJ software. The experiment was repeated three times.

#### Histology, immunohistochemistry and immunostaining

Specimens with completed decalcification were dehydrated in increasing ethanol concentrations (50%, 70%, 90%, and 100%) and embedded in paraffin for histological evaluation. Then, the resulting samples were serially cut into 5 μm thick sections using a paraffin slicer. The sections were stained with hematoxylin & eosin (H&E) and Masson trichrome staining according to standard protocols to evaluate the recovery of bone defects and new bone formation.

The paraffin sections were subjected to immunohistochemical (IHC) analysis to evaluate osteogenic differentiation and mineralization marker osteocalcin (OCN; 23,418–1-AP; Proteintech, Group, Chicago, IL, USA). Firstly, the paraffin sections were baked, placed in xylene to deparaffinise, antigenically repaired using EDTA Antigen Repair Solution (pH 9.0; ZSGB-BIO, China), eliminated endogenous peroxidase, incubated with OCN antibody (Proteintech) overnight at 4 °C, and then washed with PBS, incubated with enzyme-labeled goat-anti-rabbit IgG polymer (ZSGB-BIO) for 1 h and washed with PBS. Finally, the sections were stained with DAB chromogenic kit (ZSGB-BIO). Bone histomorphometric analyses were performed for all stained sections by the light microscope (Leica, Germany). Finally, three microscopic spans of the defects were selected and analyzed using Image J software.

The paraffin sections were subjected to immunofluorescence (IF) analysis to evaluate pyroptosis. According to the manufacturer’s instructions. And antibodies used in the experiment were anti-NLRP3 (19,771–1-AP; Proteintech) and anti-GSDMD (20,770–1-AP; Proteintech). Cell nuclei were stained with DAPI (Sigma). The images was observed using an fluorescence microscope and the immunofluorescence intensity in bone sections was quantified using Image J software.

### Statistical analysis

All experiments were performed in triplicate and all quantitative data were expressed as mean ± SD, and significant differences between multiple groups were analyzed using analysis of variance (ANOVA) method by GraphPad Prism 9.0 software. Differences were considered to be statistically significant when p < 0.05. (*P < 0.05, **P < 0.01).

## Results

### Characterization of BMSCs

**Fig. 1:**
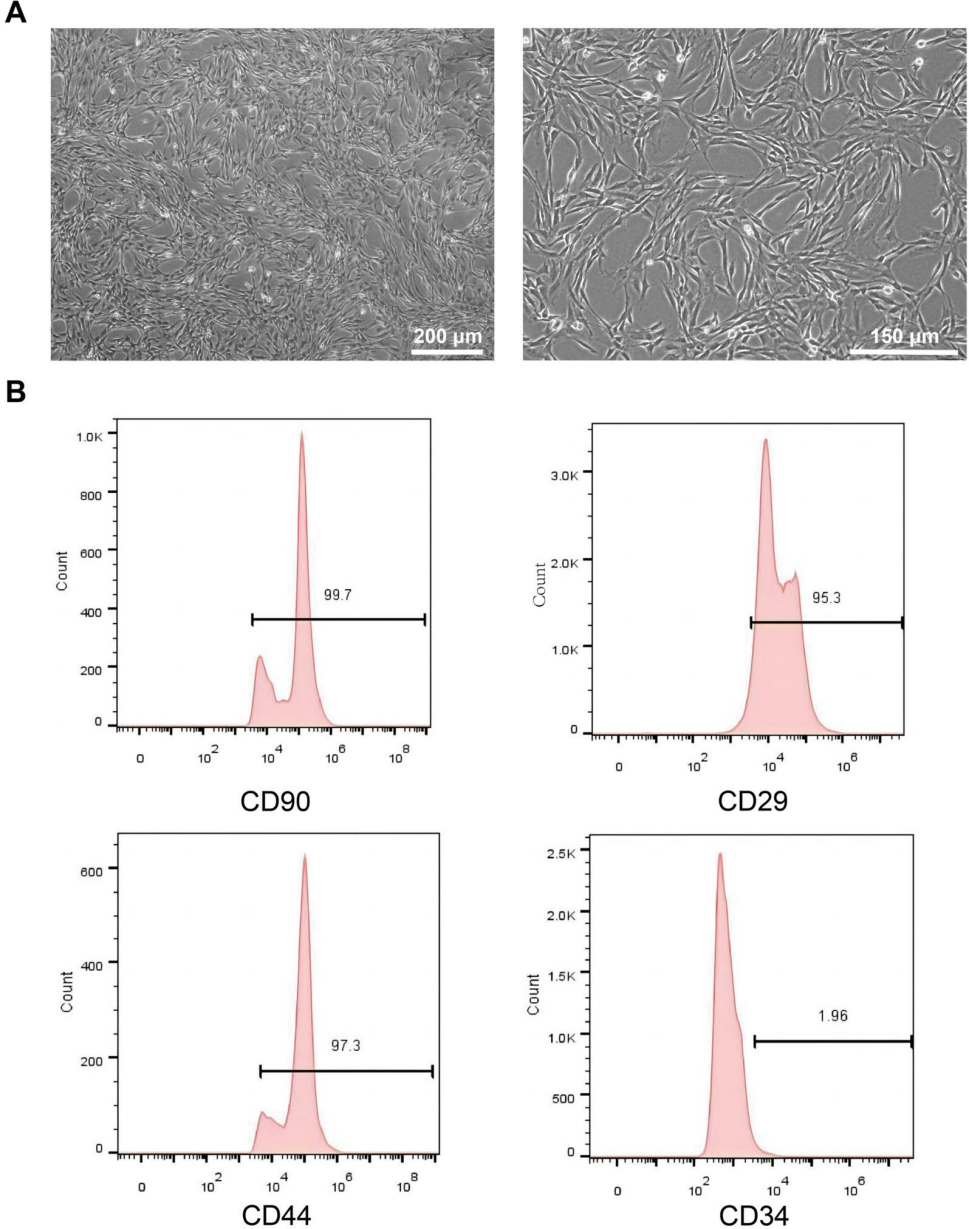
Cell morphology and identification of the BMSCs. **A** Inverted microscope image revealing the isolated BMSCs (Sale bar: 200/150 μm). **B** Flow cytometry analysis of BMSCs, and the results showed the following: CD90 ( +), CD29 ( +), CD44 ( +), and CD34 ( −)

The morphological features and molecular markers of BMSCs were identified. When BMSCs from rat were cultured to the third generation, they exhibited a fusiform and flattened morphology with a vortex-like morphology (Fig. 1A). Flow cytometry results showed that the surface of BMSCs positively expressed CD90, CD44 and CD29, but were negative for CD34. (Fig. 1B).

### Low survival of BMSCs overexpressing NGF in response to fracture inflammation is strongly associated with P75NTR-mediated pyroptosis

**Fig. 2:**
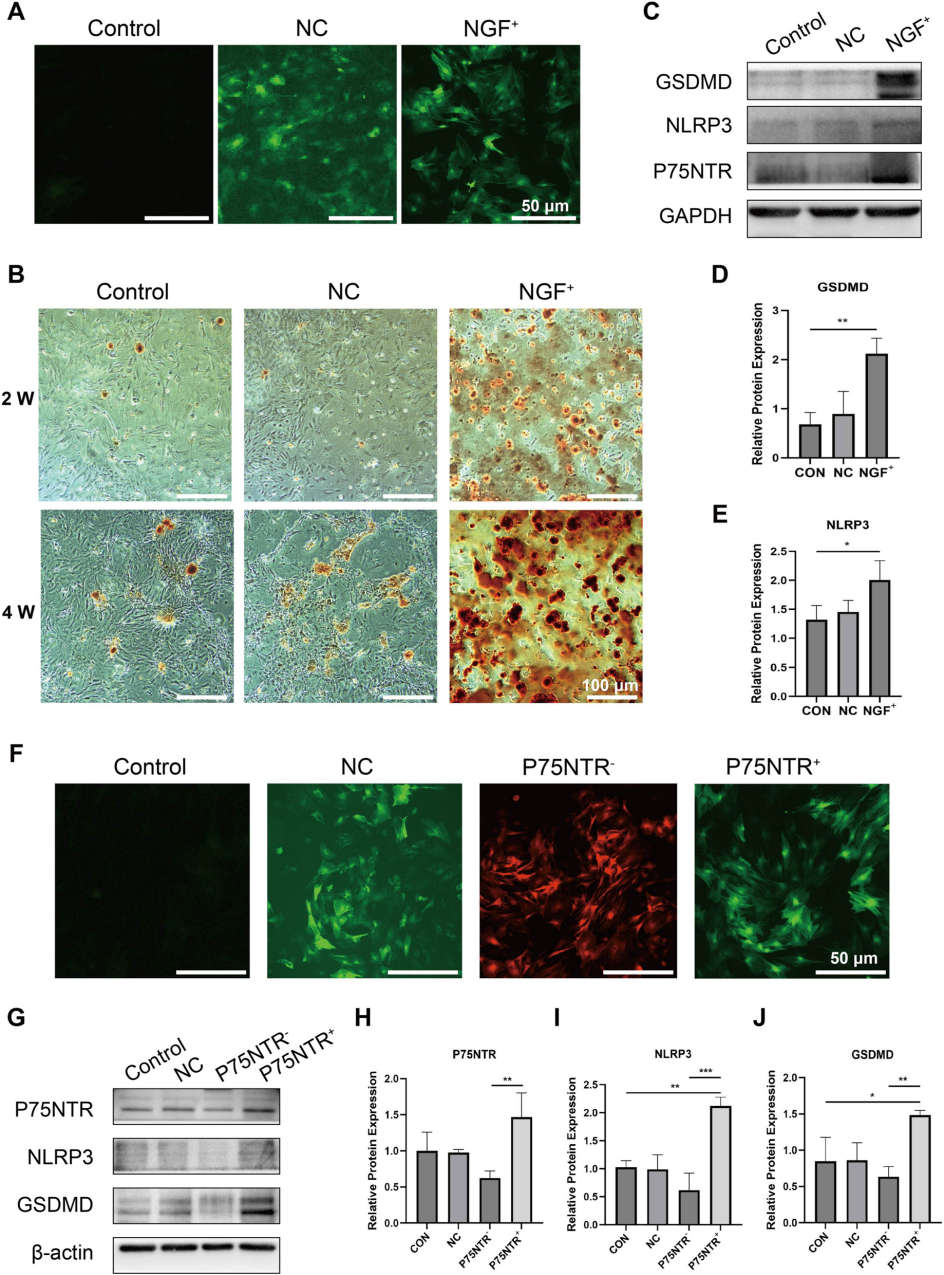
Effect of overexpressing NGF and P75NTR on BMSCs of mineralization and pyroptosis. **A** Fluorescent microscope image of each group (Sale bar: 50 μm). **B** Representative image of Alizarin red staining of BMSCs induced in osteogenic medium for 2 and 4 wk (Sale bar: 50 μm). **C-E** Protein levels of the pyroptosis-related proteins GSDMD and NLRP3, as measured by Western blotting. Corresponding uncropped full‑length gels and blots are presented in Additional file 2. **F** Fluorescent microscope image of each group (Sale bar: 50 μm). **G-J** Protein levels of P75NTR, NLRP3 and GSDMD as measured by Western blotting

After three days of lentivirus transfection, fluorescence microscopy revealed green fluorescent expression in the NGF overexpression transfection group and the negative transfection, but no fluorescent expression in the blank groups (Fig. 2A). Mineralized nodules are a hallmark of osteoblast maturation, and we assessed mineralized nodule formation by ARS staining. Figure 2B depicts the formation of calcium deposits and nodules after two and four weeks of osteogenic induction, with more calcium deposits and nodules forming in the NGF overexpression group than in the control group. Meanwhile, we confirmed that the NGF overexpression group produced cellular markers of cellular pyroptosis while promoting osteogenic differentiation of BMSCs by examining the protein expression levels of specific pyroptosis markers. GSDMD and NLRP3 are specific proteins during cellular pyroptosis[33, 34]. The Western blot results revealed that pyroptosis proteins GSDMD and NLRP3 were highly expressed in the NGF overexpression group (Fig. 2C-E).

P75NTR overexpression transfection group showed green fluorescence expression, P75NTR knockdown transfection group showed red fluorescence expression (Fig. 2F). The fluorescence expression rate of the target gene could reach approximately 70%. In addition, we detected pyroptosis protein expression levels in BMSCs by overexpression and knockdown of P75NTR. The results showed that the relative expression of pyroptosis protein in the overexpression P75NTR transfection group was significantly higher than that in the negative transfection group and the blank group, and the relative expression of pyroptosis protein in the knockout P75NTR transfection group was significantly lower than that in the negative transfection group and the blank group (Fig. 2G-J). These results suggest that the occurrence of inflammatory death in osteogenic differentiation of BMSCs overexpressing NGF may be associated with pyroptosis. Moreover, changes in P75NTR expression could regulate changes in the expression of proteins related to pyroptosis in BMSCs, and a positive correlation was observed.

### NSA can block pyroptosis in BMSCs overexpression NGF

**Fig. 3:**
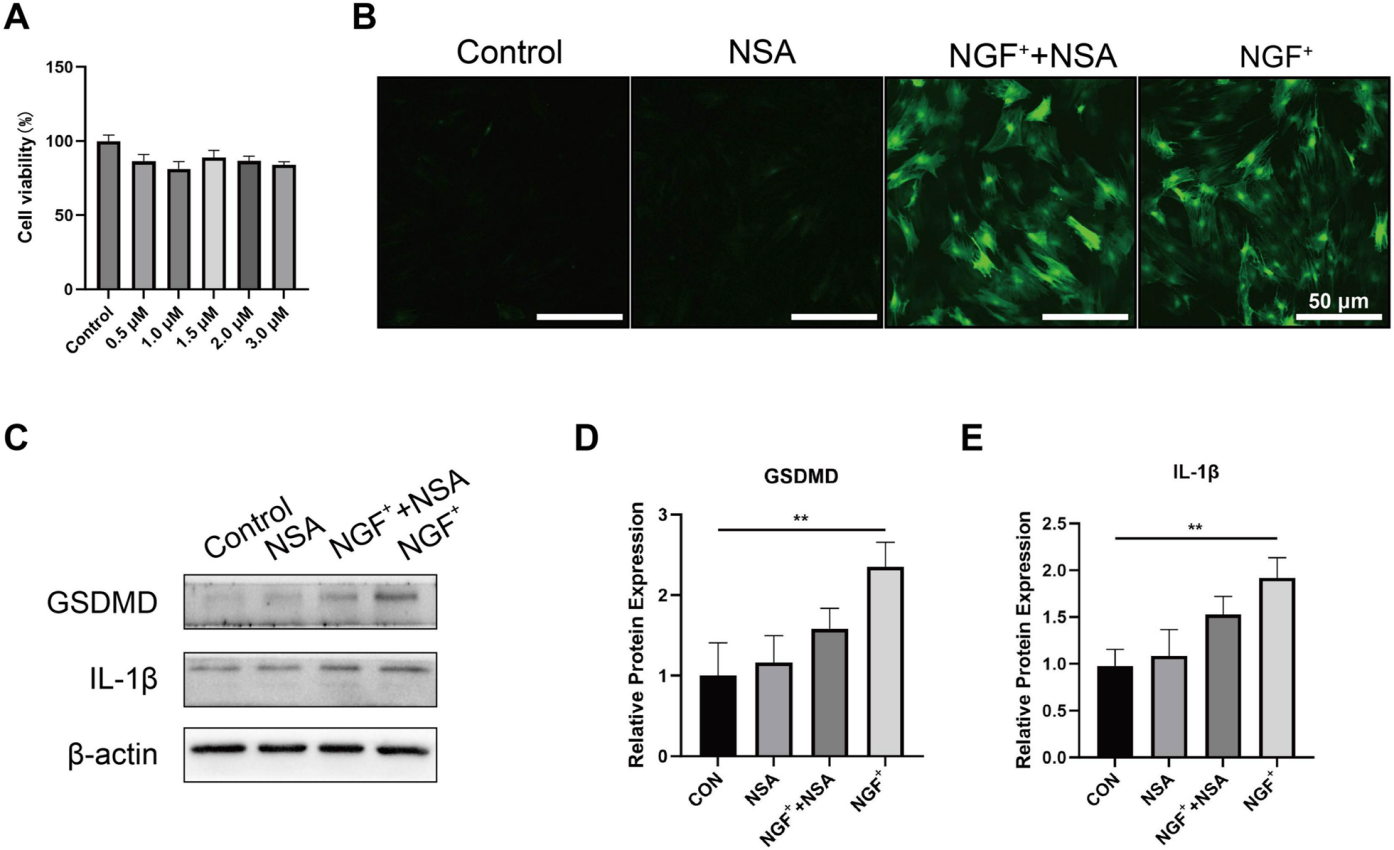
Effect of NSA and overexpressing NGF on BMSCs of pyroptosis. **A** The cell viability of BMSCs after they were treated with different concentrations of NSA for 24 h. **B** Fluorescent microscope image of each group (Sale bar: 50 μm). **C-E** Protein levels of the pyroptosis-related proteins GSDMD and IL-1β, as measured by Western blotting.  Corresponding uncropped full‑length gels and blots are presented in Additional file 2

We evaluated the cell viability of BMSCs cultured under different NSA concentrations separately to explore the role of NSA in the growth and proliferation of BMSCs. The cell viability of BMSCs cultured under an NSA concentration of 1.5 μM was higher (Fig. 3A). Three days after transfection, fluorescence microscopic presented green fluorescence expression in the NGF^+^/BMSCs and NGF^+^/BMSCs-NSA transfected groups, but no fluorescent expression was observed in the NSA and blank groups (Fig. 3B).

We confirmed the effect of NGF^+^/BMSCs, NGF^+^/BMSCs-NSA, or BMSCs-NSA on the gene expressions related to pyroptosis by examining the protein expression levels of the pyroptosis markers GSDMD and IL-1β. GSDMD and IL-1β were highly expressed in the NGF overexpression group. However, NGF^+^/BMSCs-NSA and BMSCs-NSA showed significant downregulation (Fig. 3C-E). These results suggest that pyroptosis in the differentiation of BMSCs overexpression NGF can be blocked by NSA.

### Characteristics of scaffolds

**Fig. 4:**
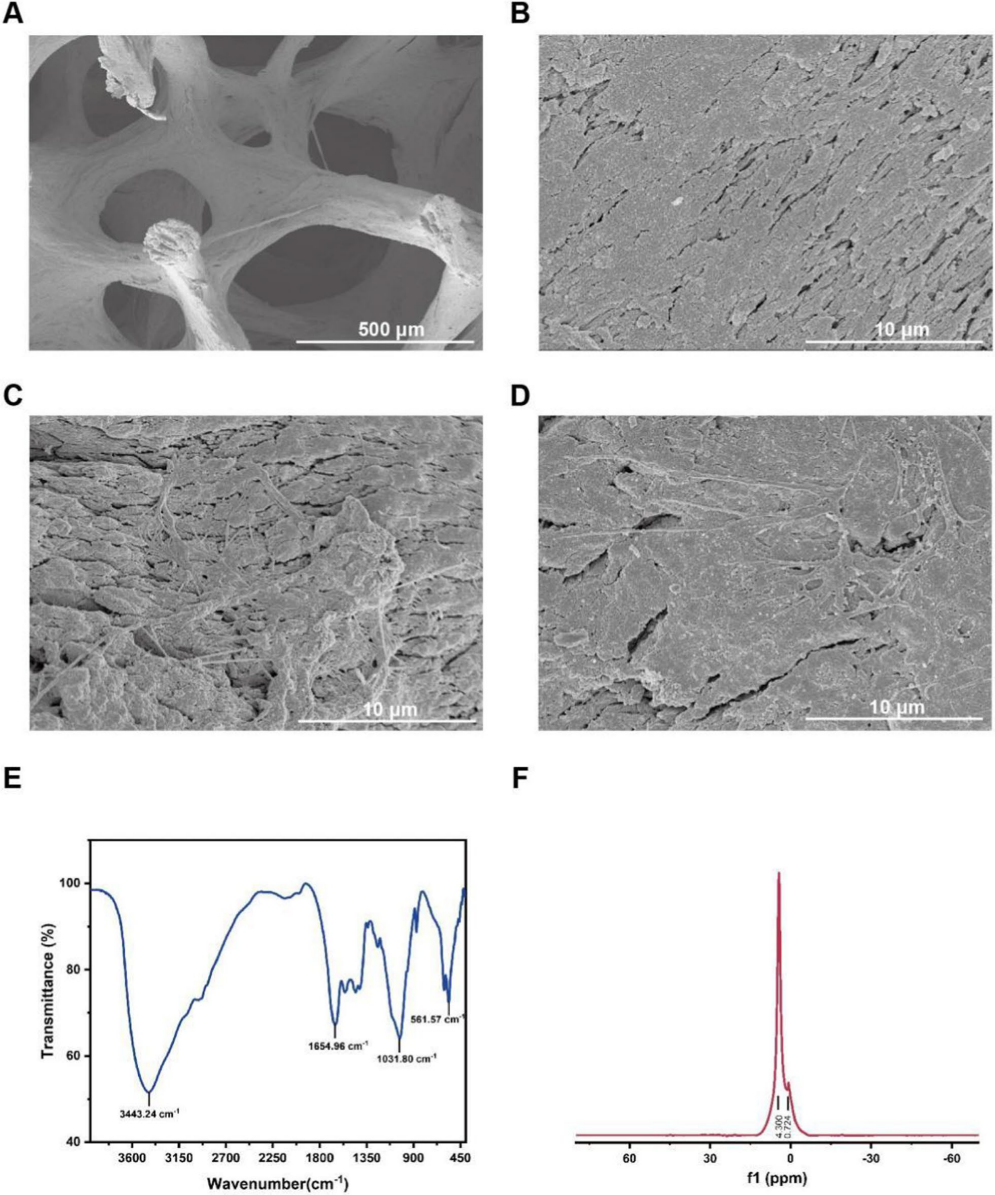
Characteristics of the scaffolds. **A,B** SEM observation of the allogenic bone scaffolds (Sale bar: 500/10 μm). **C,D** SEM observation of the BMSCs cultured on the allogenic bone scaffolds (Sale bar: 10 μm). **E** FTIR spectra of the allogenic bone scaffolds. **F** 1H NMR spectra of the allogenic bone scaffolds

SEM micrographs exhibited the surface structure of the scaffolds to be a micron-sized porous structure ranging from 200 to 500 μm, with the distribution of the pores being quite homogeneous (Fig. 4A-B). Figure 4C-D showed the SEM image of cells adhering to the scaffold 3 days after inoculation with the filopodia extending into the scaffolds, and the results showed that the scaffold had good biocompatibility. Furthermore, FTIR spectrometer of scaffolds have been shown in Fig. 4E. The telescopic vibrational peak at 3443 cm^−1^ is caused by the N–H bonds of the amine group in the scaffolds, and the peaks at 1654 cm^−1^ of the spectral band are attributed to the telescopic vibrational absorption peaks of the amides C=O. The peaks at 1031 cm^−1^ and 561 cm^−1^ have the phosphate P-O vibrational peak, and the 500–1200 cm^−1^ segment primarily reacts to the scaffold’s phosphate component. Additionally, the chemical structure of the scaffolds was determined using NMR. Figure 4F illustrates the NMR result with a peak of approximately 4.30 ppm. It suggests that the scaffold’s inorganic composition is primarily phosphate and carbonate.

### NGF^+^/BMSCs-NSA could promote early healing of SD rat bone defect

**Fig. 5:**
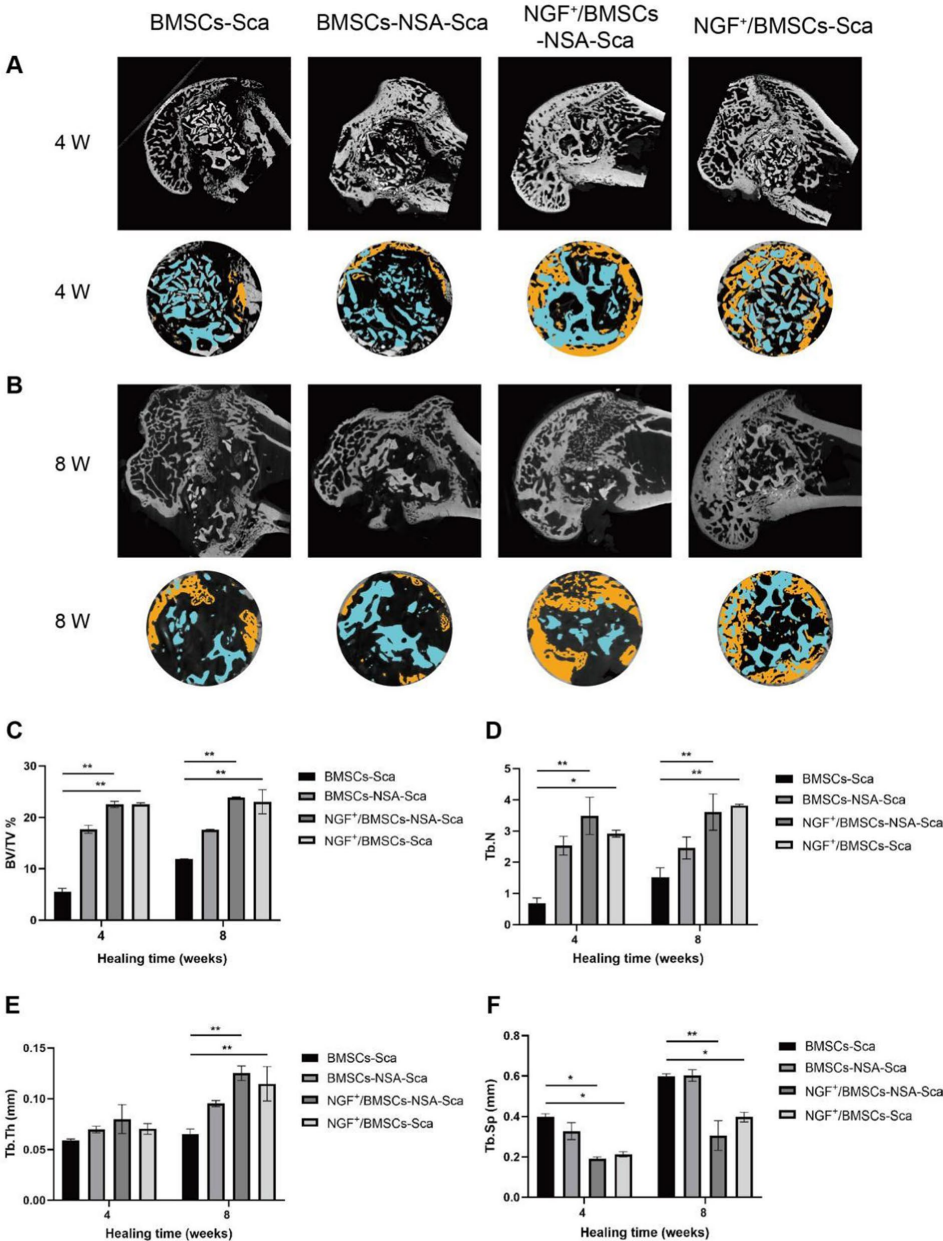
Micro-CT image analysis of femoral condylar bone regeneration. **A** Reconstructed three-dimensional micro-CT images at 4 wk after surgery. **B** Reconstructed three-dimensional micro-CT images at 8 wk after surgery. **C** Comparison of BV/TV in the bone defect area at post-operative 4 and 8 wk. **D** Comparison of Tb.N in the bone defect area at post-operative 4 and 8 wk. **E** Comparison of Tb.Th in the bone defect area at post-operative 4 and 8 wk. **F** Comparison of Tb.Sp in the bone defect area at post-operative 4 and 8 wk

To observe new bone formation within the femoral condylar defects in rats, we performed micro-CT analyses of defects implanted with NGF^+^/BMSCs, NGF^+^/BMSCs-NSA, BMSCs-NSA, and BMSCs-only allografts at four and eight weeks after surgery (Fig. 5A, B). These results confirmed the new bone formation within the defects. At each time point after surgery, dense new bone tissue was visible around the grafts in the NGF^+^/BMSCs-NSA group, whereas only a small amount of new bone formation was observed in the control group. BV/TV% within ROI was measured to assess new bone formation around the grafts, as previously described (Fig. 5C). At each postoperative time point, BV/TV was significantly higher in the NGF^+^/BMSCs-NSA group than in the control group (p < 0.01) and slightly higher than in the NGF^+^/BMSCs group. Additionally, trabecular structures were already visible at the edges of the defect in the NGF^+^/BMSCs-NSA group, whereas the middle portion of the graft had not yet completely degraded. Compared with the NGF^+^/BMSCs group, the number and thickness of bone trabeculae in the NGF^+^/BMSCs-NSA group increased, but the separation degree of bone trabeculae decreased (Fig. 5D-F). This demonstrated the osteogenic capacity of NGF^+^/BMSCs-NSA allogeneic bone constructs.

**Fig. 6:**
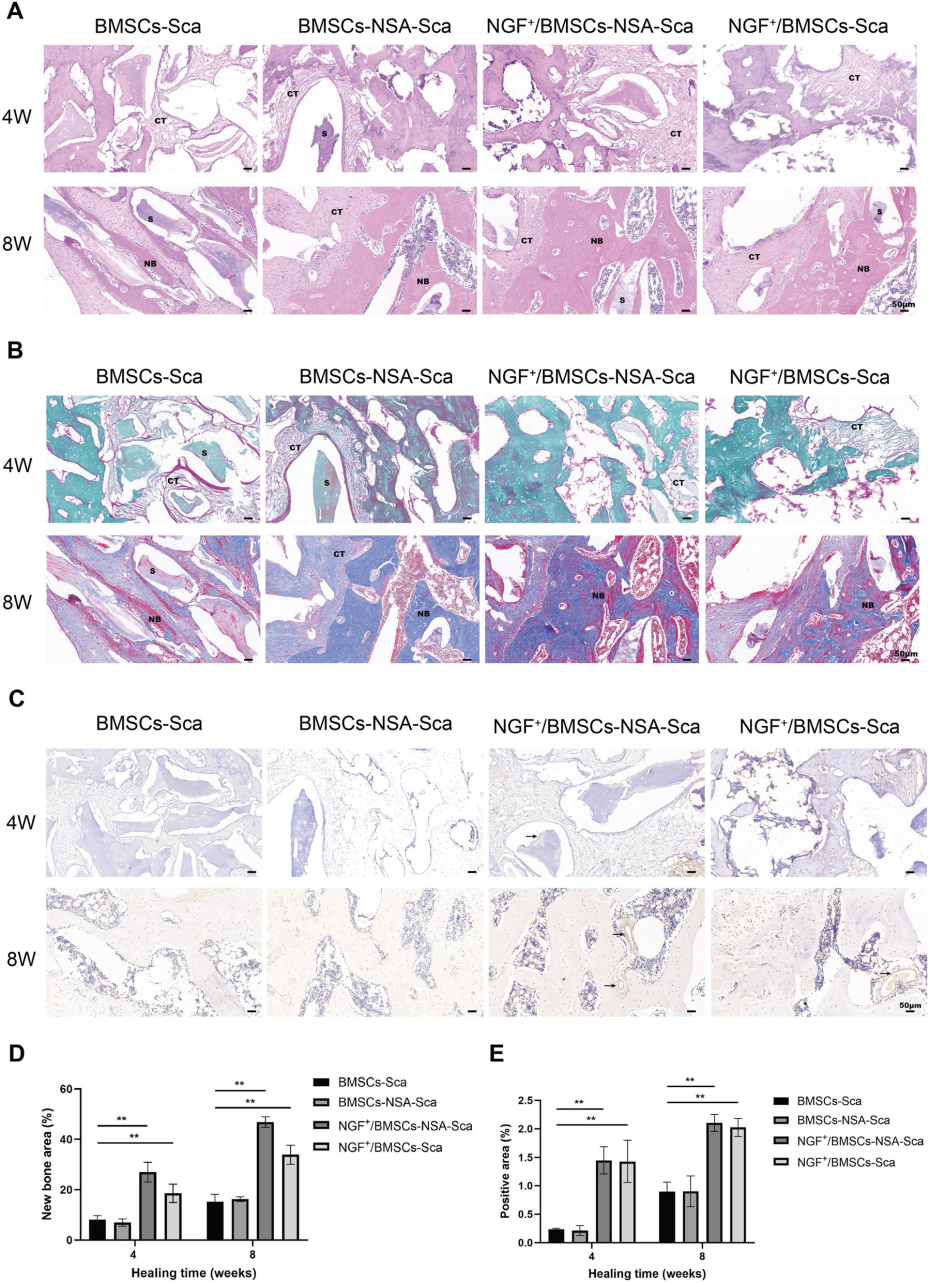
Histology and immunohistochemistry analysis. **A** new bone and osteocytes analysis by HE staining for each treatment group at 4 and 8 wk after after surgery. **B** Masson's trichrome staining at post-operative 4 and 8 wk. **C** Immunohistochemical assessment of OCN at post-operative 4 and 8 wk. Black arrow represented the positive cells. **D** The area of new bone was quantified by masson trichrome staining and is presented in the histogram. **E** The area of positive anti-OCN staining was quantified. (NB: new bone, CT: connective tissue, S: scaffold, Sale bar: 50 μm)

**Fig. 7:**
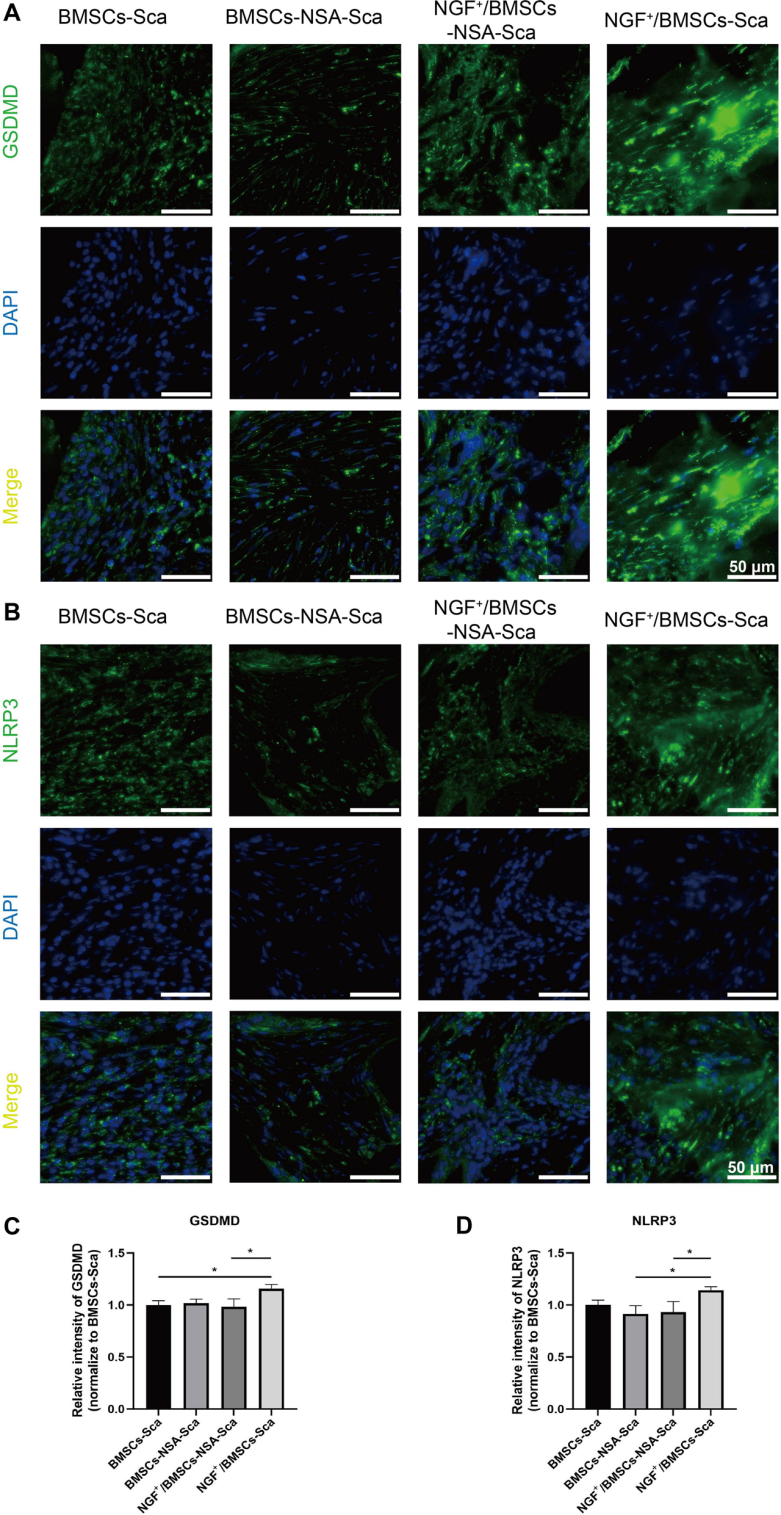
Immunofluorescence analysis. **A** Bone tissue from the SD rat femoral condylar defect at 8 wk after after surgery were stained with anti-GSDMD (green) for immunostaining analysis. **B** Bone tissue were stained with anti-NLRP3 (green) antibody for immunostaining analysis. (Sale bar: 50 μm). **C** The immunofluorescence intensity of GSDMD in bone sections was quantified and is presented in the histogram. **D** The immunofluorescence intensity of NLRP3 in bone sections was quantified

In addition, we used H&E, Masson's trichrome, immunohistochemistry and immunofluorescence to visualize the histological manifestations of bone regeneration in rat femoral condylar defects. The tissue section staining results were consistent with the imaging findings. H&E staining revealed a few osteoblasts. A small amount of neovascularization was visible at the edges of the NGF^+^/BMSCs-NSA group at 4 weeks after surgery. However, almost no new bone formation was detected in the controls. H&E staining exhibited that a small amount of new bone was visible in the control group at eight weeks after surgery. Specifically, the NGF^+^/BMSCs-NSA group displayed numerous new bones visible within the local connective tissue, few osteoblasts visible at the edge of the bone trabeculae, and the implant had a significant bone healing effect (Fig. 6A). Similarly, Masson trichrome staining displayed that numerous new bones and collagenous tissues were visible in the NGF^+^/BMSCs-NSA group (Fig. 6B and D). Immunohistochemical staining analysis presented that the immune response intensity to OCN was significantly higher in the group treated with NGF^+^/BMSCs-NSA scaffold constructs than in the control group after four and eight weeks after surgery (Fig. 6C and E). Furthermore, immunofluorescence result revealed the expression of pyroptosis-related signals GSDMD and NLRP3 in group NGF^+^/BMSCs-Sca was higher than that in control group, while the expression of pyroptosis in group NGF^+^/BMSCs-NSA-Sca was down-regulated (Fig. 7A-D). In conclusion, these results demonstrate that combining NGF^+^/BMSCs-NSA on allogeneic bone scaffolds can improve the osteogenic capacity to repair bone defects.

### NGF^+^/BMSCs-NSA could involve bone regeneration via inhibiting cell pyroptosis

**Fig. 8:**
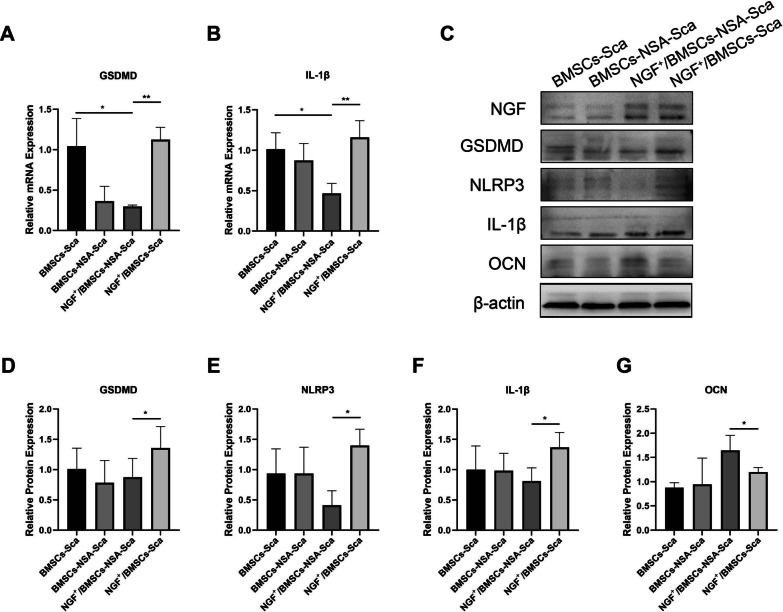
NSA and Over-expression of NGF regulated pyroptosis and Osteogenic related genes at 4w after surgery. **A, B** mRNA levels of the pyroptosis-related genes GSDMD and IL-1β at 4 weeks after surgery, as determined by real-time PCR. **C-G** Protein levels of the proteins NGF, GSDMD, NLRP3, IL-1β and OCN at 4 weeks after surgery, as measured by Western blotting. Corresponding uncropped full‑length gels and blots are presented in Additional file 2

**Fig. 9:**
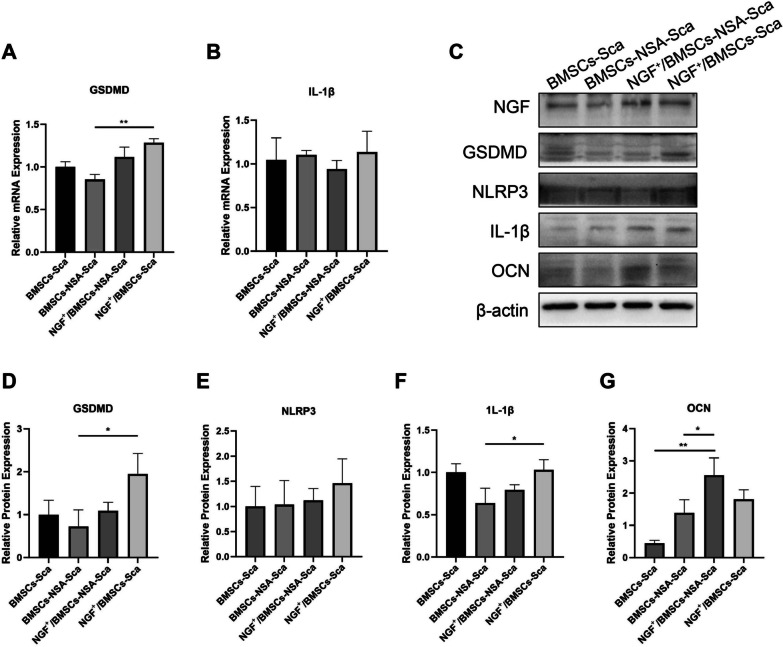
NSA and Over-expression of NGF regulated pyroptosis and Osteogenic related genes at 8w after surgery. **A, B** mRNA levels of the pyroptosis-related genes GSDMD and IL-1β at 8 weeks after surgery, as determined by real-time PCR. **C-G** Protein levels of the proteins NGF, GSDMD, NLRP3, IL-1β and OCN at 8 weeks after surgery, as measured by Western blotting. Corresponding uncropped full‑length gels and blots are presented in Additional file 2

We measured the GSDMD and IL-1β mRNA levels at the defects in rats at four and eight weeks after surgery, respectively, to examine the effects of NGF^+^/BMSCs-NSA and NGF^+^/BMSCs on the death-related gene expressions (Figs. 8A, B and 9A, B). Figure 8A, B illustrate the results at four weeks after surgery. The pyroptosis-related signals GSDMD and IL-1β mRNA levels were significantly up-regulated in the NGF^+^/BMSCs group, whereas the GSDMD and IL-1β mRNA levels were significantly down-regulated in the NGF^+^/BMSCs-NSA group (p < 0.01). Similarly, the GSDMD and IL-1β mRNA levels were slightly down-regulated in the NGF^+^/BMSCs-NSA group than in the NGF^+^/BMSCs group at eight weeks after surgery (Fig. 9A, B). These results demonstrated that NSA inhibited the pyroptosis-associated genes, GSDMD, and IL-1β mRNA levels and attenuated the pyroptosis effect from NGF overexpression.

As previously described, we verified the effects of NGF^+^/BMSCs-NSA and NGF^+^BMSCs on pyroptosis-related protein expression using Western blotting. The results exhibited that the pyroptosis-related protein expression levels of GSDMD, IL-1β, and NLRP3 were enhanced in the NGF^+^/BMSCs group at four weeks after surgery, while pyroptosis protein levels were reduced in the NGF^+^/BMSCs-NSA group (Fig. 8C-F). The osteoblastic protein expression levels of OCN were enhanced in the NGF^+^/BMSCs-NSA group (Fig. 8G). Protein blotting exposed the same at eight weeks after surgery (Fig. 9D-G). These results demonstrated that NSA inhibited the pyroptosis-related protein expression levels of GSDMD, IL-1β, and NLRP3 and promoted the osteogenic effects of NGF overexpression. The blocking effect of NSA mentioned above is verified again.

## Discussion

In this study, we attempted to define the low survival of BMSCs overexpressing NGF in response to fracture inflammation and to improve it to promote regeneration of bone defect repair. Initially, we used lentiviral vectors loaded with overexpressed NGF genes to transfect BMSCs to determine their relationship with P75NTR and pyroptosis. Nerve Growth Factor induces osteogenic differentiation of various cells to promote fracture healing. For example, Yang et al. investigated the mechanism of action of NGF in the mouse embryonic osteogenic precursor cell line MC3T3-E, and the results showed that NGF promoted the proliferation and osteogenic differentiation of MC3T3-E while increasing the expression of BMP-2 [35]. Additionally, NGF promotes angiogenesis and trophic regeneration of skeletal sensory nerves. And vascular and nerve regeneration can significantly promote the process of bone repair. Recent studies have demonstrated that NGF stimulates the migration of skeletal cells during early bone repair and exerts various cell-specific functions [36]. Indeed, Our previous experiments indicated that cell survival was relatively low after NGF overexpression transfection of BMSCs, and P75NTR silencing combined with NGF overexpression double gene co-transfection of BMSCs demonstrated a good osteogenic capacity [37].

P75NTR is involved in various cell responses, such as apoptosis, survival, migration, and axonal growth. P75NTR has been reported to activate NF-κB signaling, up-regulate MMP-9 and VEGF expression, induce inflammation, and disrupt the blood–brain barrier in astrocytes [38]. Inhibition of P75NTR expression is known to improve the survival of neuro differentiated BMSCs [39] and enhance osteogenic differentiation of rat BMSCs [20]. Here, we found that overexpression of NGF can lead to the increase of P75NTR, and the change of P75NTR expression can regulate the expression of pyrogen related proteins in BMSCs with positive correlation. Consequently, these results support our suspect that the reduced survival of BMSCs overexpressing NGF in the fracture inflammatory response may be associated with P75NTR-induced pyroptosis. Combined with our previous studies, here our focus is to improve the pyroptosis response during the transplantation of BMSCs overexpressing NGF during the healing process of bone defects, in order to increase the survival rate of BMSCs for accelerated repair of bone defects.

In order to clarify the low survival of BMSCs overexpressing NGF during the healing process of bone defects, we selected NLRP3, IL-1β and GSDMD, which are key factors in the process of pyroptosis. Activation of NLRP3 inflammatory vesicles is the key to pyroptosis. This was reported by Wang et al., they found that NLRP3 assembles into NLRP3 inflammatory vesicles upon sensing certain pathogens, and then cuts GSDMD, promotes the secretion of IL-1β and IL-18, and causes inflammatory cell death [40]. Moreover, GSDMD has been reported to be closely related to pyroptosis and inflammation, and GSDMD is cleaved to produce N-GSDMD fragments, forming pores that increase membrane permeability, leading to pyroptosis and IL-1β release [41]. Both classical and non-classical pathways of pyroptosis require activation of GSDMD to lead to the generation of pyroptosis. Therefore, in this study, NLRP3 and GSDMD were selected as entry points for the verification of pyroptosis. This provides a target selection scheme for inhibiting pyroptosis and solving the problem of low cell survival rate in bone defect healing.

In addition, we also found that the pyroptosis of BMSCs overexpressing NGF during fracture inflammation can be blocked by NSA. During pyroptosis, NSA prevents pyroptosis by inhibiting the aggregation of long chains of GSDMD on the cell membrane. Furthermore, Zhang et al. showed that NSA could affect the mRNA and protein expression of pyroptosis related genes, inhibit the secretion of IL-6, TNF-α and IL-1β by osteoblasts, and they also found that NSA promoted the proliferation and differentiation of osteoblasts by inhibiting of NLRP3/caspase-1/GSDMD pyroptosis pathway [42]. Therefore, we selected NSA as a pyroptosis inhibitor. NSA can inhibit the pyroptosis reaction of BMSCs overexpressing NGF during the healing of bone defects in order to improve the survival rate of BMSCs. Our findings highlight the potential of NSA in inhibiting the pyroptosis of NGF^+^/BMSCs, making it a therapy to improve the low survival of BMSCs overexpressing NGF. Notably, the therapeutic NSA concentrations used in this study did not exhibit cytotoxic effects on cells.

In vitro having demonstrated that NSA can inhibit the pyroptosis of NGF^+^/BMSCs in fracture inflammation, we proceeded to the final and most important aim of this study which focused on determining the ability of NGF^+^/BMSCs-NSA-Sca to promote bone regeneration and defect repair in vivo, especially in a rat model of femoral condylar defect. In this study, allograft bone was used as a scaffold to implant BMSCs loaded with overexpressed NGF. Allograft bone as a graft has strong mechanical strength, good biocompatibility, and bone conductivity, and making it a better choice for repairing bone defects [43]. Consistent with previous studies [44], allogeneic bone scaffolds could effectively support the BMSCs attachment and expansion in our experiments. We constructed novel grafts to repair rats bone defects by combining NGF-overexpressed BMSCs with allograft bone and adding pyroptosis inhibitors. Additionally, previous studies have used a rat femoral condylar defect model to assess bone regeneration and defect repair in vivo [45]. Another study revealed that the critical defect size that could not spontaneously be repaired was 3.5/5.0 mm [46]. Therefore, we evaluated the osteogenic capacity of NGF^+^/BMSCs-NSA combined with allograft bone scaffolds in a surgical model of bone defects in rats femoral condyles with a diameter of 4.5 mm and a depth of 5.0 mm. Micro-CT analysis and histological evaluation revealed that NGF^+^/BMSCS-NSA scaffolds significantly enhanced new bone formation compared to scaffolds with BMSCs alone. Furthermore, WB and PCR results showed that NGF^+^/BMSCs-NSA-Sca group had relatively low pyroptosis genes and relatively high osteogenic genes. This demonstrated that NGF^+^/BMSCs combined with NSA can increase the transplantation survival rate of BMSCs by inhibiting pyroptosis, and thus participate in bone regeneration to accelerate the repair of bone defects. Overall, the results suggest that the potential of the NGF^+^/BMSCs-NSA-Sca system as a novel therapy for bone defects.

Through the above studies, we believe that NGF^+^/BMSCs-NSA-Sca is helpful to develop a new theoretical basis for the treatment of bone defects. However, the specific molecular mechanisms and pathway of pyroptosis in BMSCs overexpressing NGF are not yet clear, and whether it is related to the P75NTR-mediated NF-κB pathway. Therefore, specifically, our future work will focus on elucidate the relationship between pyroptosis in BMSCs overexpressing NGF and the NF-κB pathway in the inflammatory environment of fractures.

## Conclusion

In conclusion, reduced survival of BMSCs overexpressing NGF in localised inflammation of fractures is closely related to P75NTR-induced pyroptosis, which can be inhibited by the use of NSA, and the use of BMSCs overexpressing NGF combined with allogeneic bone scaffolds and NSA as grafts accelerates bone defect repair and regeneration. Overall, we anticipate that the synergistic effect of BMSCs, scaffolds, and NGF during bone tissue regeneration contributes to bone tissue engineering material development with excellent properties.

### Supplementary Information

**Additional file 1**: Supplementary message of the BMSCs.

**Additional file 2**: The original image of the immunoblotting.

## Data Availability

The data that support the findings of this study are available from the corresponding author on reasonable request.

## Abbreviations

NGF: Nerve growth factor
BMSCs: Bone marrow mesenchymal stem cells
NSA: Necrosulfonamide
SD: Sprague–Dawley
P75NTR: Nerve growth factor receptor
GSDMD: Gasdermin D
IL-1β: Interleukin 1beta
OCN: Osteocalcin
NLRP3: NOD-like receptor thermal protein domain associated protein 3
L-DMEM: Low glucose dulbecco’s modified Eagle medium
PBS: Phosphate-buffered saline
ARS: Alizarin Red S
PVDF: Polyvinylidene fluoride
FTIR: Fourier transform infrared spectroscopy
1H NMR: Hydrogen proton nuclear magnetic resonance spectroscopy
SEM: Scanning electron microscopy
PBS: Phosphate-buffered saline
qRT-PCR: Quantitative reverse transcription polymerase chain reaction
Micro-CT: Micro-Computed Tomography
BV/TV: Bone volume per tissue volume
Tb.N: Trabecular number
Tb.Th: Trabecular thickness
Tb.Sp: Trabecular separation
BCA: Bicinchoninic acid
H&E: Hematoxylin and eosin
MTT: Thiazolyl blue
IHC: Immunohistochemical
BMP-2: Bone morphogenetic protein 2
MMP-9: Matrix metalloprotein-9
VEGF: Vascular endothelial growth factor
NF-κB: Nuclear transcription factor-κB
