# Supplementary material for: Acceleration of bone repairation by BMSCs overexpressing NGF combined with NSA and allograft bone scaffolds
Source: Stem Cell Res Ther. 2024 Jul 2;15:194. doi: 10.1186/s13287-024-03807-z (PMC11218317; doi:10.1186/s13287-024-03807-z)
Supplement: Supplementary file 1 — Additional file 1: Supplementary message of the BMSCs. [file 13287_2024_3807_MOESM1_ESM.pdf]

产品检测报告

OriCell®SD 大鼠骨髓间充质干细胞

产品货号：RASMX-01001

We help you discover life

基本信息

产品名称	OriCell®SD 大鼠骨髓间充质干细胞
货号	**RASMX-01001**
批号	230906C41
供体信息	雄性
冻存代次	**P2**
保存条件	液氮（-196℃）

检测标准及结果

检测项目			检测结果	检测标准	结论
常规检测	细菌、真菌		阴性	阴性	合格
	支原体		阴性	阴性	合格
	内毒素		≤10EU	≤10EU	合格
鉴定检测	复苏存活率		87.27%	≥80%	合格
	活细胞数		1.79×10^{6}	$\geq1\times10^{6}$	合格
	细胞复苏贴壁率		98.55%	≥80%	合格
	生长状态		形态呈长梭性，呈极性排列，群体倍增时间为 17.64h	形态为长梭形，呈极性排列；群体倍增时间≤ 72 h	合格
	分化能力		可分化为成脂、成骨、成软骨细胞	经定向诱导，可分化为成骨细胞、脂肪细胞、成软骨细胞	合格
	表面标记分子	**CD90**	98.61%	≥70%	合格
		**CD34**	0.22%	≤5%	合格
		**CD45**	0.11%	≤5%	合格
		**CD44**	99.67%	≥70%	合格
		**CD11b/c**	0.19%	≤5%	合格
		**CD29**	90.95%	≥70%	合格

细胞生长状态

细胞增殖能力

选取对数期（1-3 天）数据进行计算，得出细胞群体倍增时间为 17.64h。

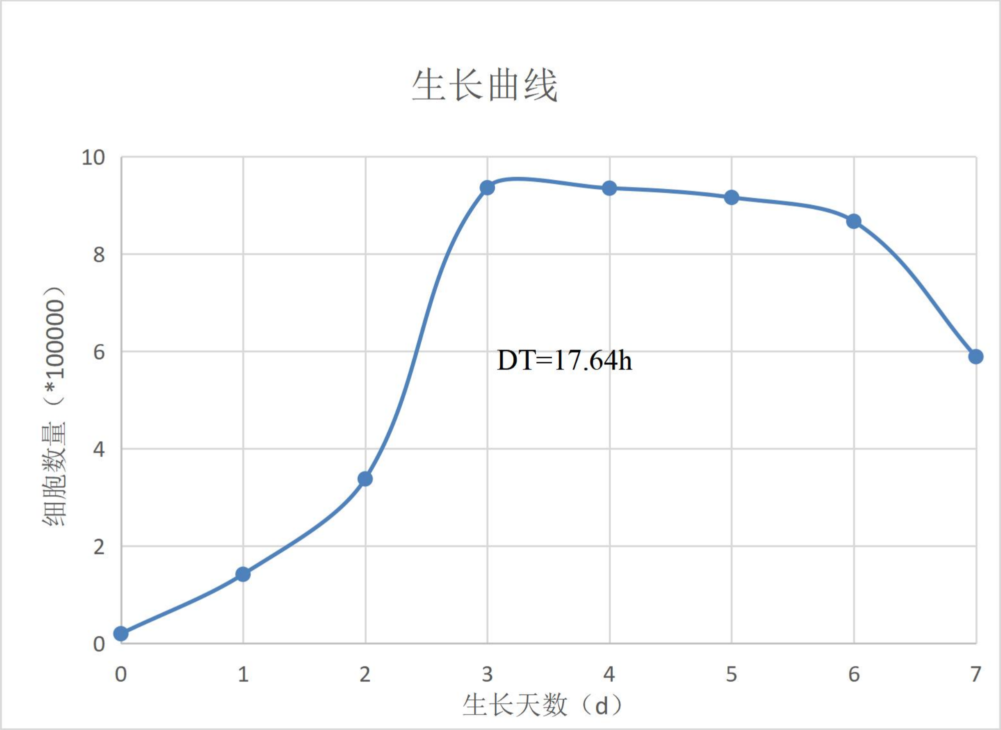

细胞传代能力

细胞均呈长梭形生长，极性良好，立体感良好。经 5 次传代后，细胞仍较有活力。

本批次细胞 Pn+1 代生长 72h 后的形态

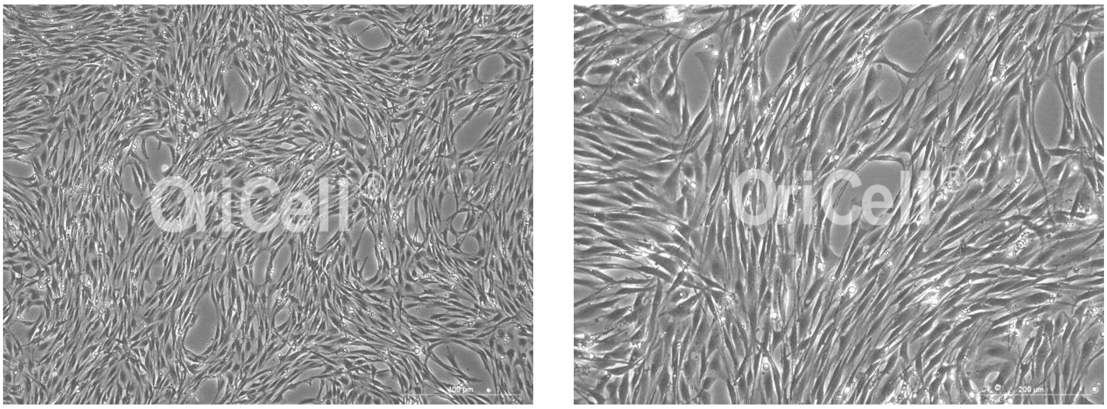

本批次细胞 Pn+3 代生长 24h 后的形态

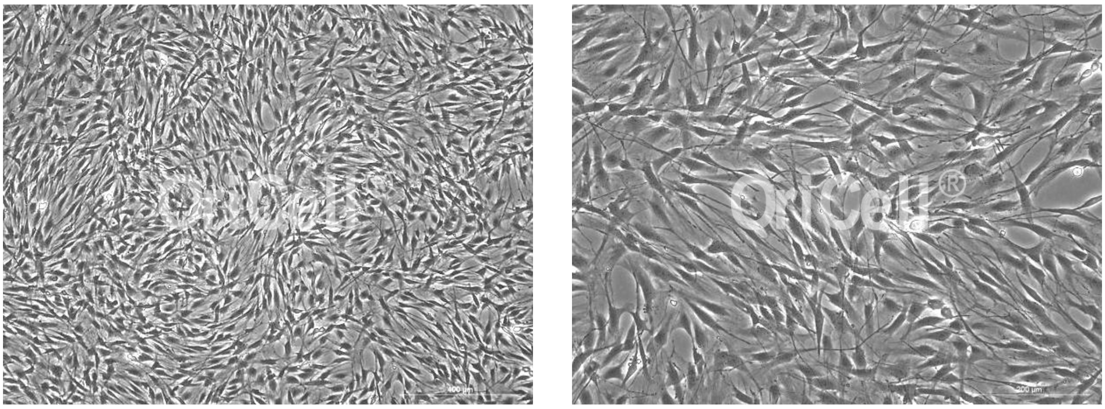

本批次细胞 Pn+5 代生长 24h 后的形态

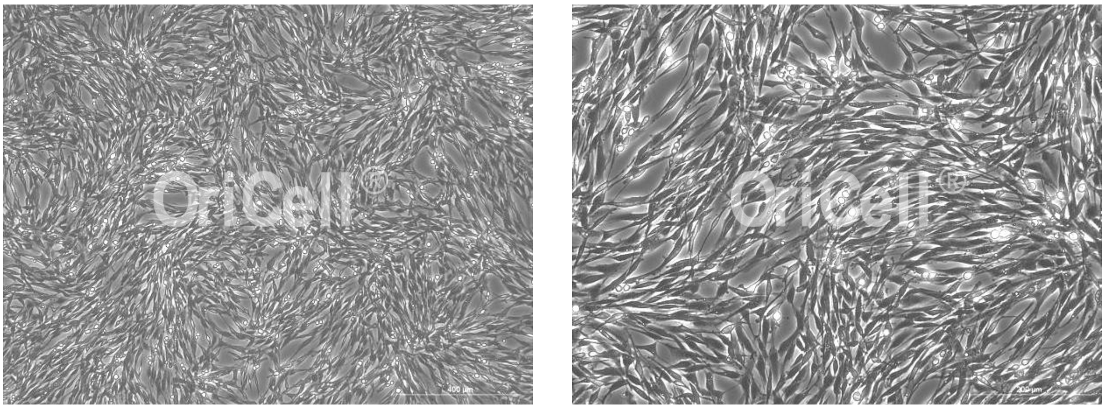

诱导分化能力

成脂诱导分化

细胞汇合度约达 90%时，加入间质干细胞成脂诱导液，诱导成功后，进行油红 O 染色，可见被染为红色的较标准的脂滴。

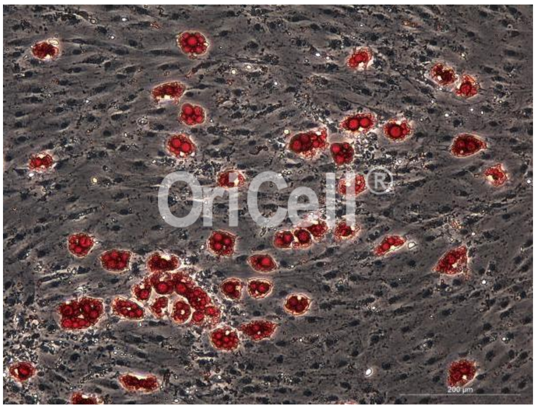

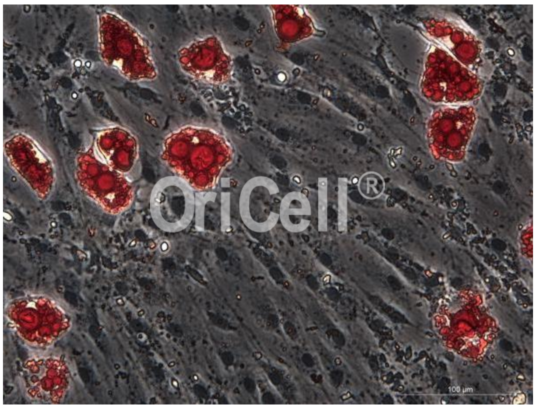

成骨诱导分化

细胞汇合度约达 70%时，加入间质干细胞成骨诱导液，诱导成功后，进行茜素红染色，茜素红与类骨质结合，形成同心圆状的深红色小结节。

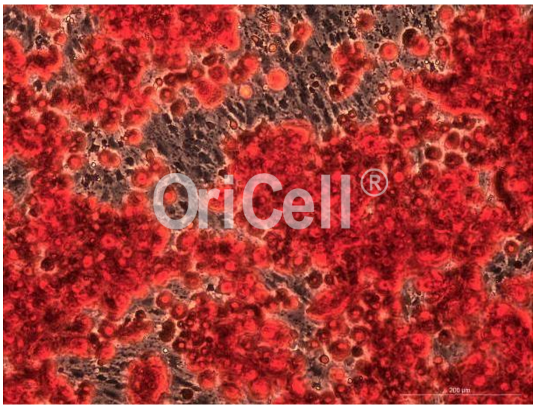

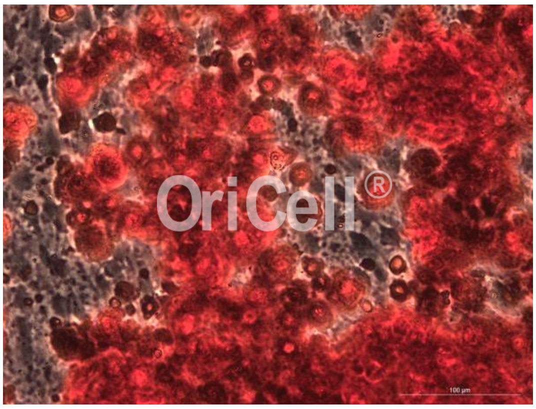

成软骨诱导分化

细胞经团块培养，间质干细胞软骨诱导液诱导培养，细胞从贴附于离心管底部的扁平细胞团逐渐变成细胞球，诱导成功后，细胞团变圆，表面变得光滑。

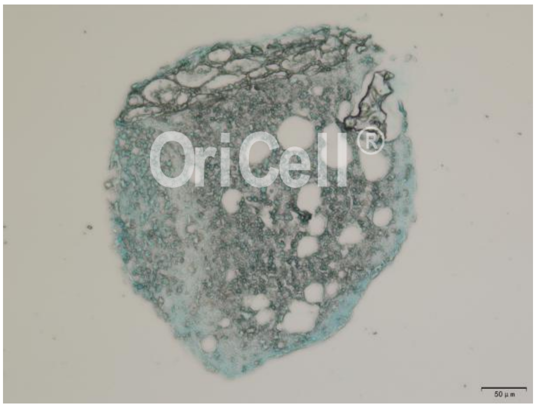

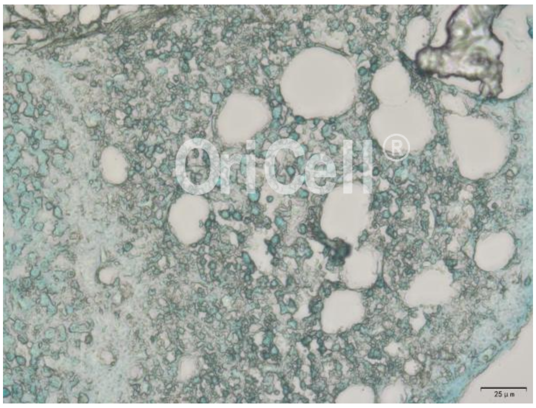

表面分子流式检测

数据详情

Mouse IgG1, κ Isotype Control Antibody

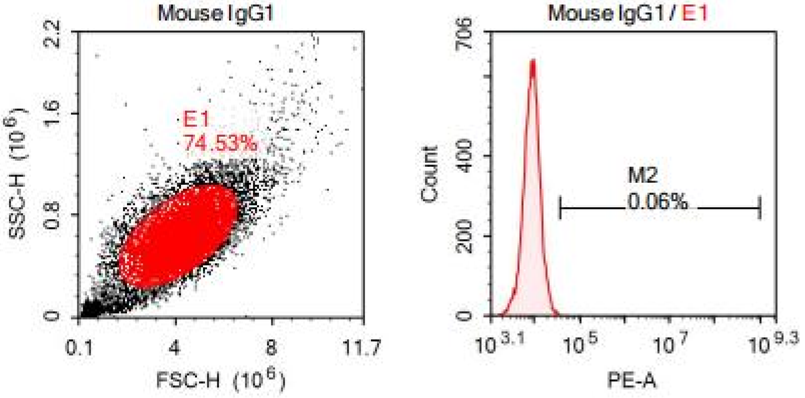

Anti-rat CD90 antibody

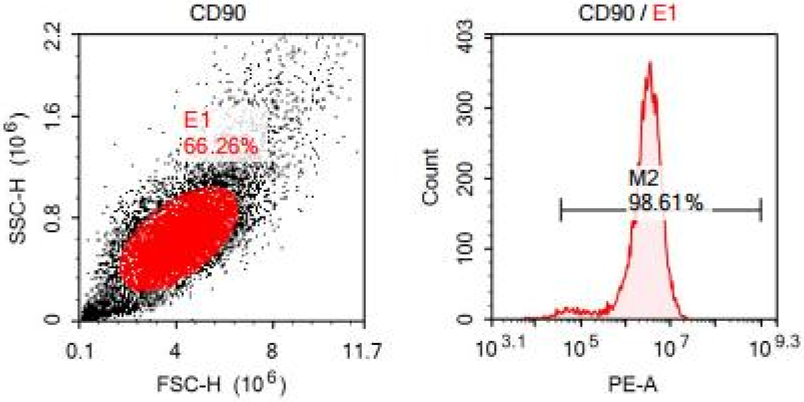

Anti-rat CD34 antibody

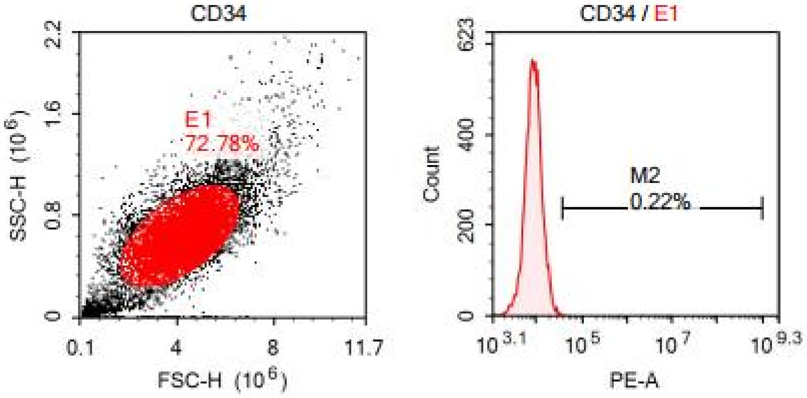

Anti-rat CD45 antibody

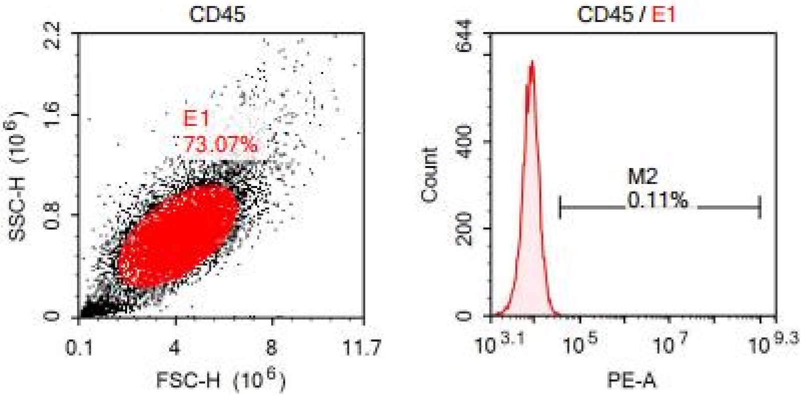

Anti-rat CD44 antibody

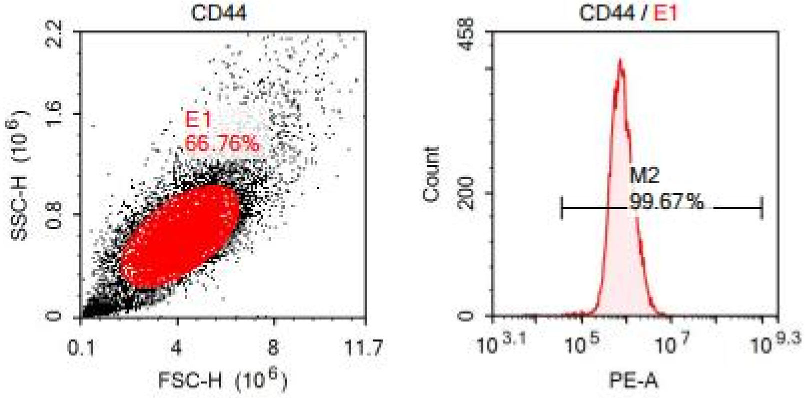

Mouse IgG2a , κ Isotype Control Antibody

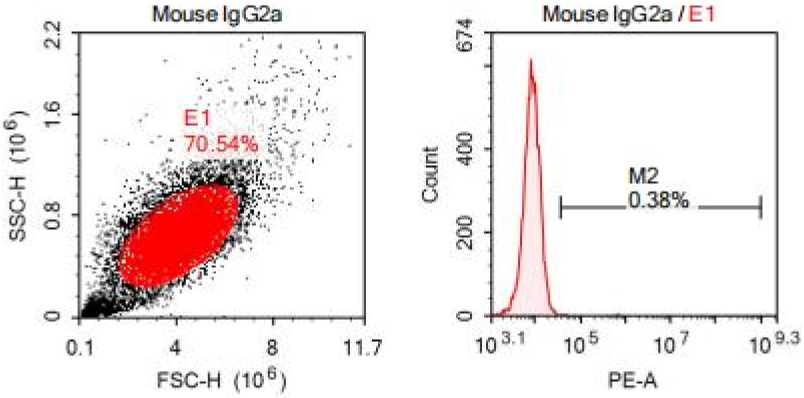

Anti-rat CD11b/c antibody

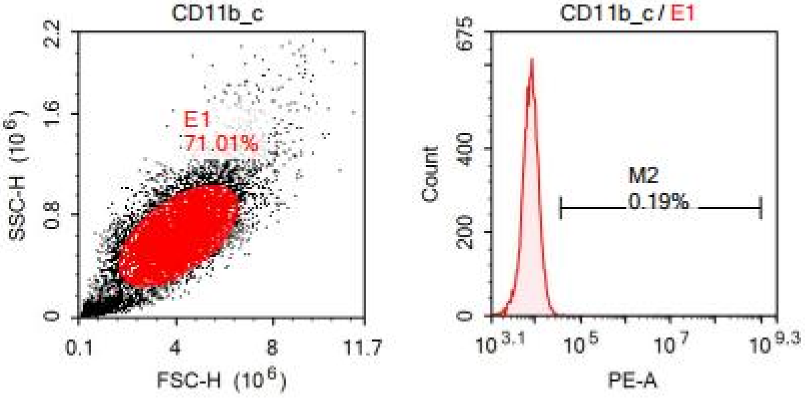

Hamster IgG, κ Isotype Control Antibody

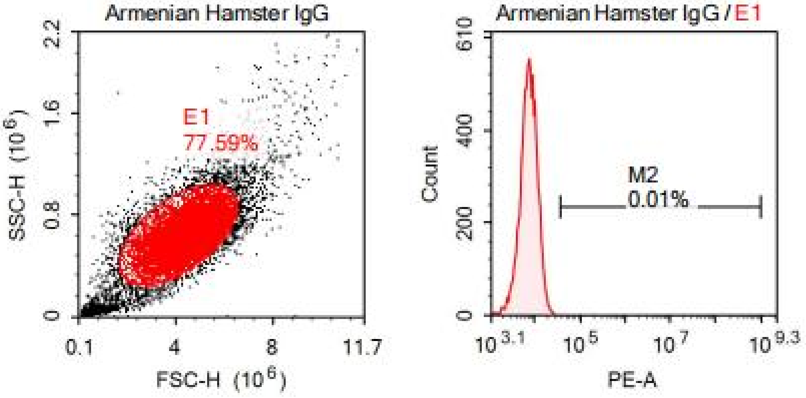

Anti-mouse/rat CD29 antibody

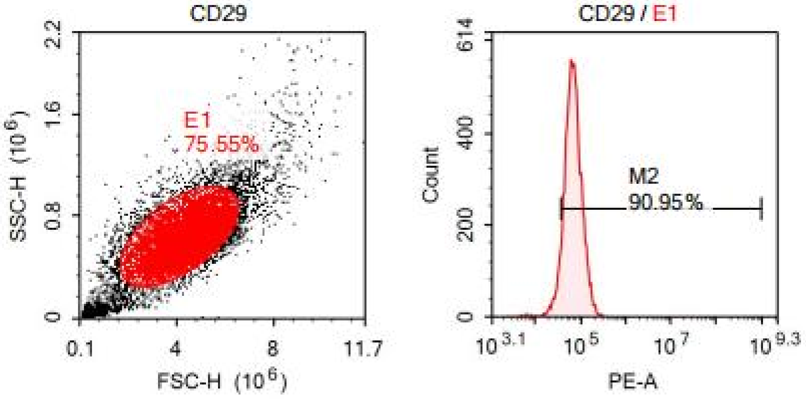

结论

本品经检测符合各项指标要求，准予放行。

检验人员：Nancy

审核人员：

赛业（广州）生物科技有限公司保留OriCell®细胞培养产品技术文件的所有权利。

没有赛业（广州）生物科技有限公司的书面许可，本文件的任何部分，

不得改编或转载用作其他商业用途。
