# Supplementary material for: Acceleration of bone repairation by BMSCs overexpressing NGF combined with NSA and allograft bone scaffolds
Source: Stem Cell Res Ther. 2024 Jul 2;15:194. doi: 10.1186/s13287-024-03807-z (PMC11218317; doi:10.1186/s13287-024-03807-z)
Supplement: Supplementary file 2 — Additional file 2: The original image of the immunoblotting. [file 13287_2024_3807_MOESM2_ESM.pdf]

Full-length blots are presented in here, the samples derive from the same experiment and that blots were processed in parallel

Fig. 2C

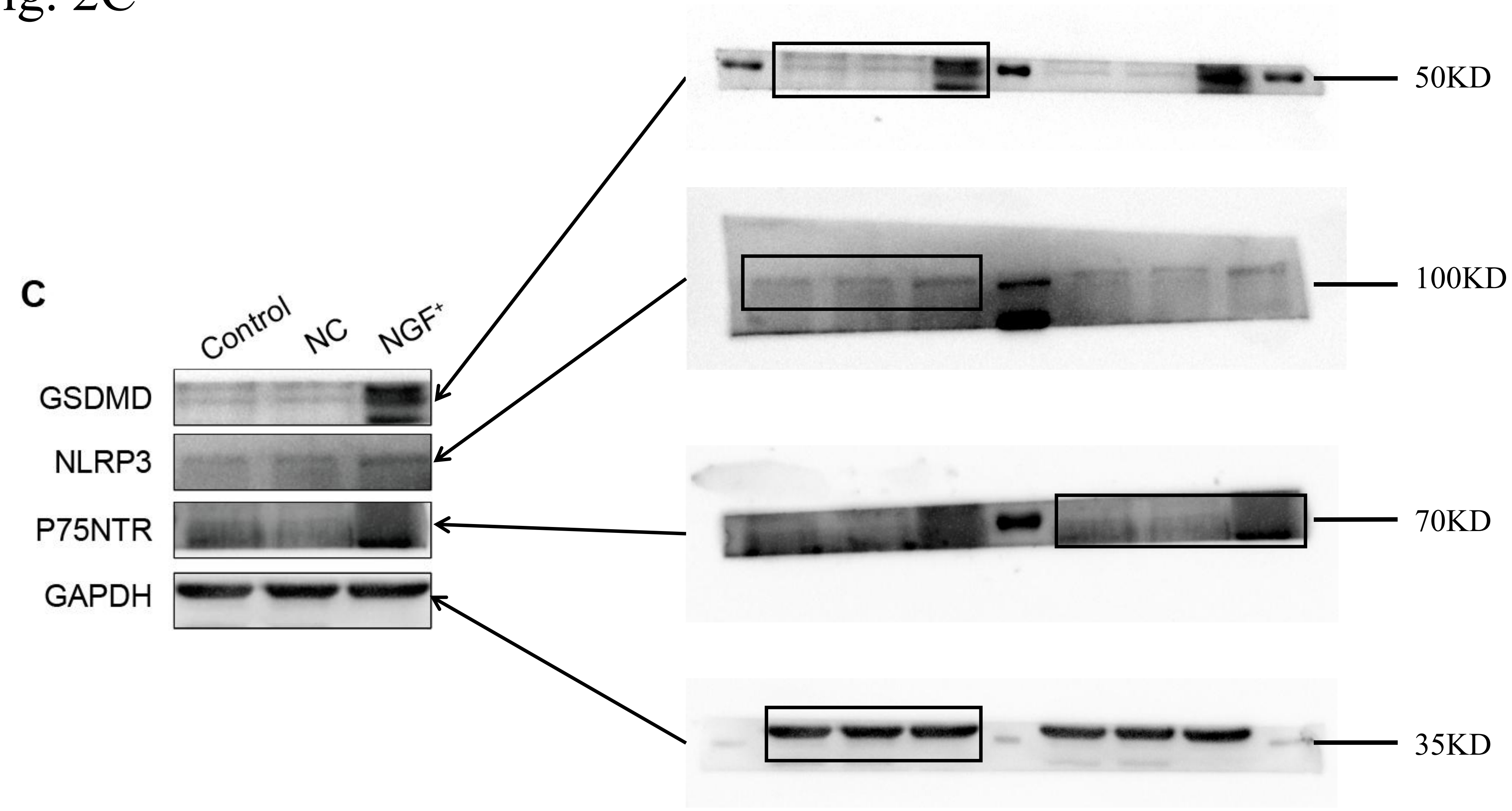
C
Control
NC
NGF+
GSDMD
NLRP3
P75NTR
GAPDH
50KD
100KD
70KD
35KD

Fig. 2G

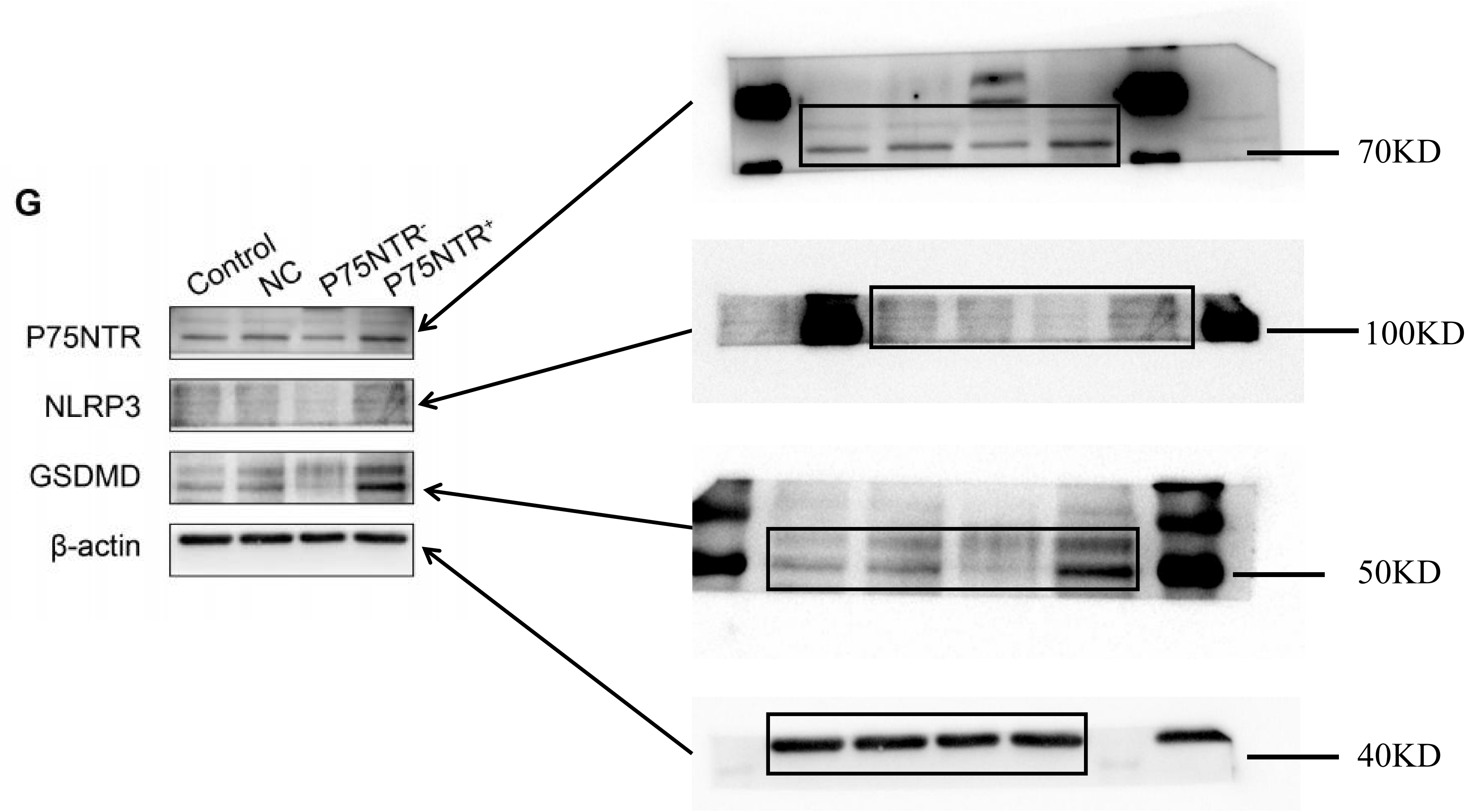
G
Control
NC
P75NTR-
P75NTR+
P75NTR
NLRP3
GSDMD
β-actin
70KD
100KD
50KD
40KD

Fig. 3C

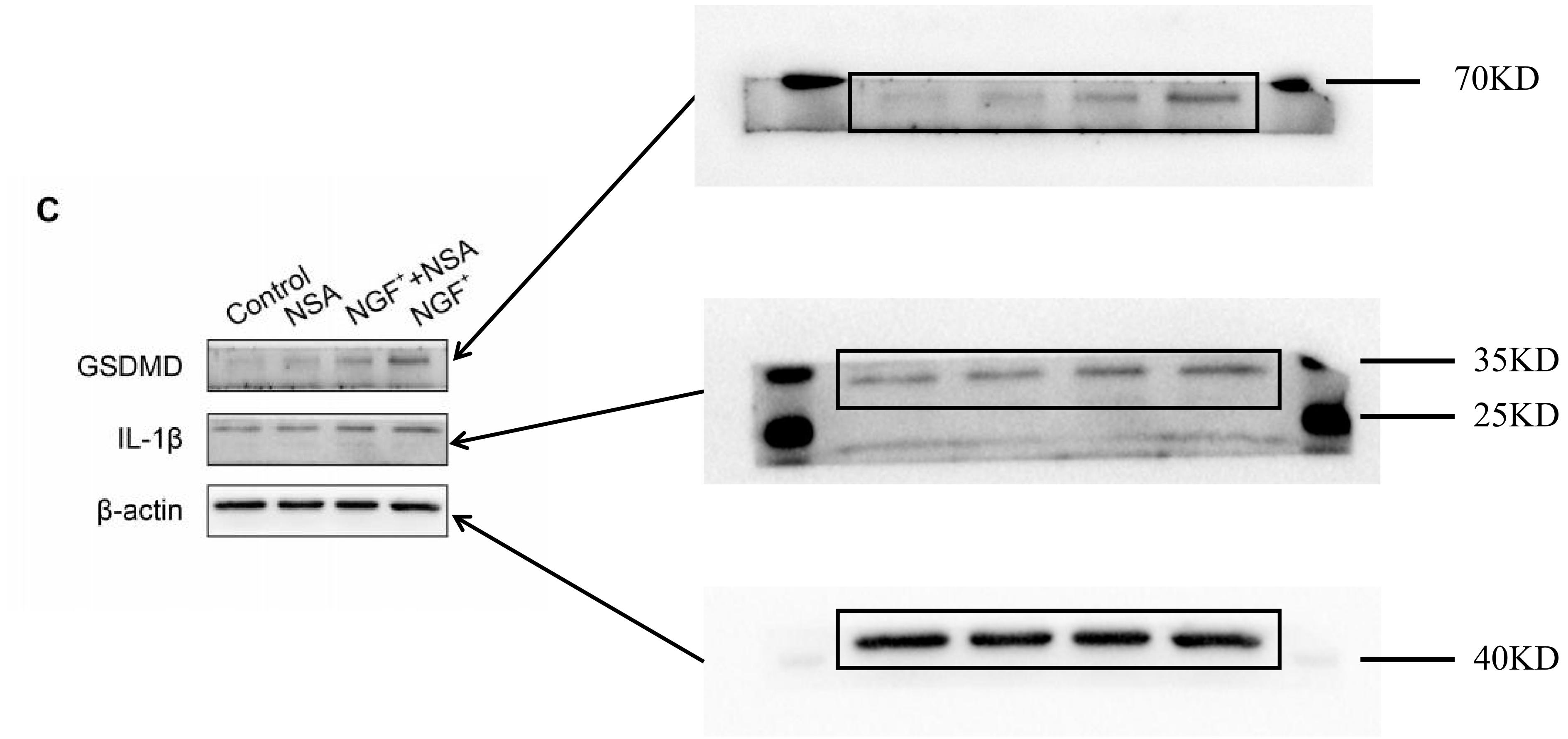
C
Control
NSA
NGF⁺+NSA
NGF⁺
GSDMD
IL-1β
β-actin
70KD
35KD
25KD
40KD

Fig. 8C

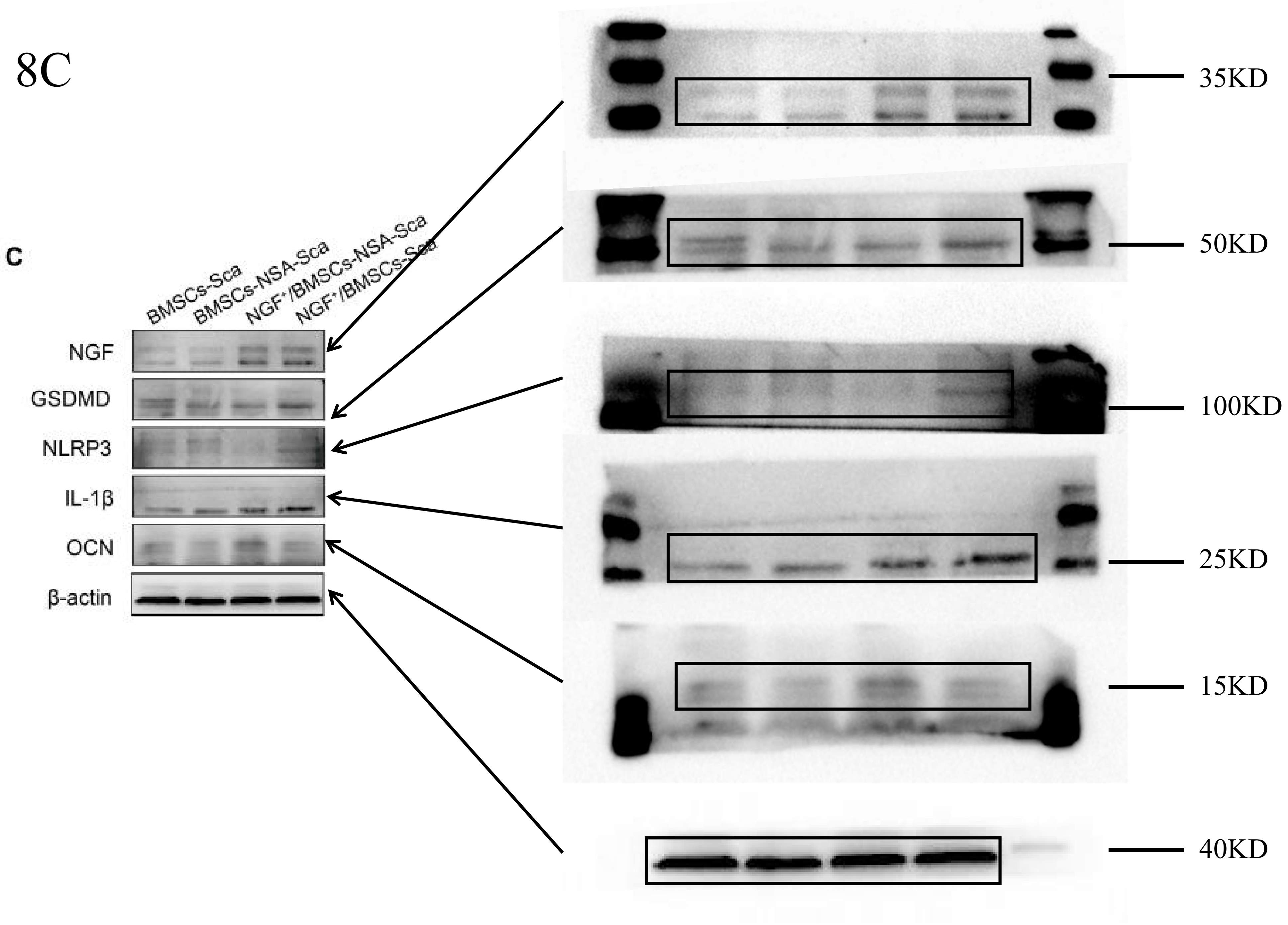
C
BMSCs-Sca
BMSCs-NSA-Sca
NGF⁺/BMSCs-NSA-Sca
NGF⁺/BMSCs-Sca
NGF
GSDMD
NLRP3
IL-1β
OCN
β-actin
35KD
50KD
100KD
25KD
15KD
40KD

Fig. 9C

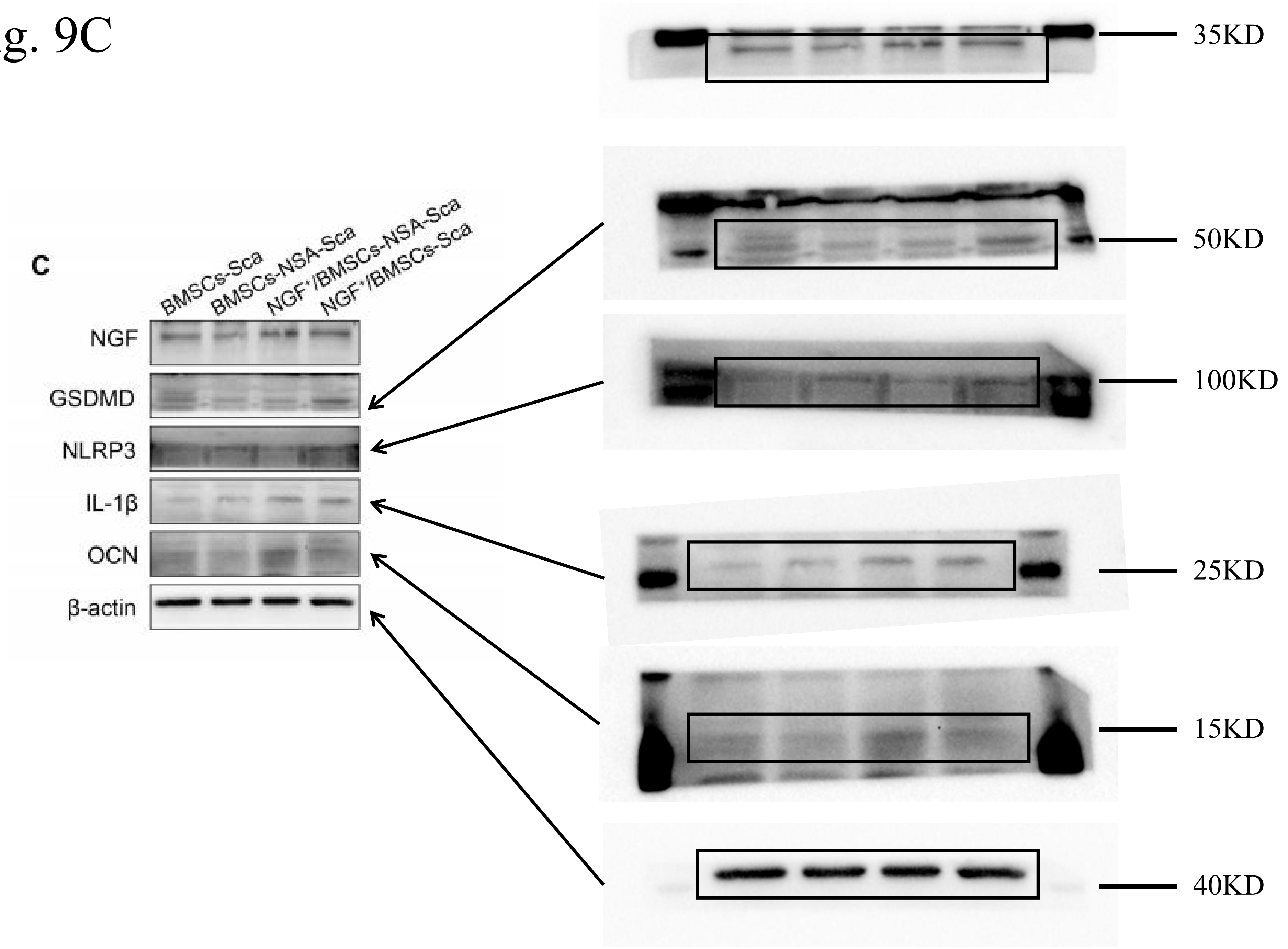
C
BMSCs-Sca
BMSCs-NSA-Sca
NGF+/BMSCs-NSA-Sca
NGF+/BMSCs-Sca
NGF
GSDMD
NLRP3
IL-1β
OCN
β-actin
35KD
50KD
100KD
25KD
15KD
40KD
